# Supplementary material for: miR-125b Upregulates miR-34a and Sequentially Activates Stress Adaption and Cell Death Mechanisms in Multiple Myeloma
Source: Mol Ther Nucleic Acids. 2019 Mar 13;16:391–406. doi: 10.1016/j.omtn.2019.02.023 (PMC6479071; doi:10.1016/j.omtn.2019.02.023)
Supplement: Document S1. Tables S1 and S2 and Figures S1–S8 [file mmc1.pdf]

OMTN, Volume *16*

## Supplemental Information

# miR-125b Upregulates miR-34a and Sequentially Activates Stress Adaption and Cell Death Mechanisms in Multiple Myeloma

**Gabriella Misso, Mayra Rachele Zarone, Angela Lombardi, Anna Grimaldi, Alessia Maria Cossu, Carmela Ferri, Margherita Russo, Daniela Cristina Vuoso, Amalia Luce, Hiromichi Kawasaki, Maria Teresa Di Martino, Antonella Virgilio, Agostino Festa, Aldo Galeone, Giuseppe De Rosa, Carlo Irace, Massimo Donadelli, Alois Necas, Evzen Amler, Pierosandro Tagliaferri, Pierfrancesco Tassone, and Michele Caraglia**

## Table S1. Mutational analysis of MM cells using Ion Reporter Browser.

### A

| Genes | Locus | Genotype | Type | Location | Amino acid change | Coding | Exon | p-value | Variant effect | Allele Source |
|---|---|---|---|---|---|---|---|---|---|---|
| MET | chr7:116411990 | C/T | SNV | exonic | p.Thr1010Ile | c.3029C>T | 14 | 0.00001 | missense | Hotspot |
| BRAF | chr7:140453132 | T/A | SNV | exonic | p.Lys601Asn | c.1803A>T | 15 | 0.00001 | missense | Hotspot |
| TP53 | chr17:7578448 | G/G | INDEL | exonic | p.Ala161fs | c.481delG | 5 | 0.00001 | frameshift deletion | Novel |
| TP53 | chr17:7578449 | T/T | SNV | exonic | p.Ala161Thr | c.481G>A | 5 | 0.00001 | missense | Hotspot |

### B

| Genes | Locus | Genotype | Type | Location | Amino acid change | Coding | Exon | p-value | Variant effect | Allele Source |
|---|---|---|---|---|---|---|---|---|---|---|
| CSDE1 | chr1:115258747 | CC/TC | SNV | Exonic, downstream | p.Gly12Asp | c.35G>A | 2 | 0.00001 | missense | Hotspot |
| PTEN | chr10:89685268 | A/C | SNV | splicesite_5 | | | 3 | 0.00001 | | Hotspot |
| TP53 | chr17:7578368 | CACCATCGCTATCTGAGCAGCGCTCATGGTGGGGGCAGCGCCTCACA/CACCATCGCTATCTGAGCAGCGCTCATGGTGGGGGCAGCCCTCACA | INDEL | splicesite_3 | | | 5 | 0.00001 | | Novel |
| TP53 | chr17:7578407 | GC/CC | SNV | exonic | p.Arg175Gly | c.523C>G | 5 | 0.00001 | missense | Hotspot |
| TP53 | chr17:7579470 | CGG/CGC | SNV | exonic | p.Pro72Arg | c.215C>G | 4 | 0.00001 | missense | Novel |

### C

| Genes | Locus | Genotype | Type | Location | Amino acid change | Coding | Exon | p-value | Variant effect | Allele Source |
|---|---|---|---|---|---|---|---|---|---|---|
| ERBB4 | chr2:212589802 | A/C | SNV | exonic | p.Phe247Cys | c.740T>G | 6 | 0.00001 | missense | Novel |
| PIK3CA | chr3:178936091 | G/A | SNV | exonic | p.Glu545Lys | c.1633G>A | 10 | 0.00001 | missense | Hotspot |
| EGFR | chr7:55242482 | CATCTCCGAAAGCCAACAAGGAAAT/TATCTCCGAAAGCCAACAAGGAAAT | SNV | exonic | p.Thr751Ile | c.2252C>T | 19 | 0.00001 | missense | Hotspot |
| KRAS | chr12:25398284 | | SNV | exonic | p.Gly12Ala | c.35G>C | 2 | 0.00001 | missense | Hotspot |
| TP53 | chr17:7577085 | | SNV | exonic | p.Glu285Lys | c.853G>A | 8 | 0.00001 | missense | Hotspot |

A: U266 cell line; B: SKMM-1 cell line; C: RPMI 8226 cell line; SNV: single nucleotide variants; INDEL: deletion; Variant effect : effect of the variant on the coding sequence; Hotspot: variant overlaps a hotspot file; Novel: not known variant.

**Table S2. TP53 variants analysis.**

| | cDNA description | Protein description | Exon number | Effect | TA Class |
|---|---|---|---|---|---|
| **U266** | c.481G>A | p.A161T | 5-exon | missense | Partially functional |
| **U266** | c.481delG | p.A161fs | 5-exon | Frameshift | NA |
| **SKMM-1** | c.215C>G | p.P72R | 4-exon | missense | NA |
| **SKMM-1** | c.523C>G | p.R175G | 5-exon | missense | Non-functional |
| **RPMI 8226** | c.853G>A | p.E285K | 8-exon | missense | Non-functional |

TA Class: transactivation activities; NA: Not Available

**Supplementary Figure legends**

**Figure S1. Evaluation of the transfection efficiency.** Flow cytometric analysis of transfection efficiency in MM cell lines (A) as fluorescence intensity. B) Analysis, by qPCR, of miR-125b expression levels in MM cell lines transfected with miR125b. Values are mean ± standard deviation (SD), n = 3.

**Figure S2. Cell viability modulation after miR-125b mimic and its modified analogs transfection.** (A-C) MTS assay of MM cell lines transfected with 100 nM of miR-125b, miR-NC, miR-125b-Omet (Omet), miR-125b-LNA (LNA) or miR-125b-2'F (F). MM cell lines U266 (A), SKMM-1 (B) and RPMI 8226 (C) were analyzed after 24h and 48h from transfection. CTR = untreated cells used as control. Each experiment was repeated at least three times and the data are shown as a mean ± standard deviation (SD).

**Figure S3. Evaluation of the transfection efficiency.** Analysis, by qPCR, of miR-125b expression levels in U266 cells 48h after transfection with miR125b or its modified analogues. Values are mean ± standard deviation (SD), n = 3.

**Figure S4. Densitometric analysis of Western Blot from Figure 3C/D.** U266 cells transfected with 100 nM of miR-125b, miR-NC, miR-125b-Omet (Omet), miR-125b-LNA (LNA) or miR-125b-2'F (F) were collected for Western blotting analysis after 24h and 48 h from transfection. Densitometric analysis of IL6-R (A), cRaf (B), Akt (C), Bcl-2 (D), Mcl1 (E), STAT3 (F), pSTAT3 (G), EIF5A (H), 4EBP1 (I), p4EBP1 (L) and p53 (M) bands was performed with ImageJ analysis tool. Each column reflects the relative amount of the protein as a ratio of each protein band relative to the corresponding loading control. * $p \leq 0.05$; ** $p \leq 0.01$; *** $p \leq 0.001$ compared to miR-NC group.

**Figure S5. Apoptosis evaluation by FACS analysis.** U266 cell line was transfected with miR-125b, miR-NC or miR-125b-Omet at a final concentration of 100 nM for 24h (A) and 48h (B). After double staining with PI and Annessin V, as described in “Materials and Methods”, cells were analyzed by flow cytometric analysis. The data are representative of three different experiments that have always yielded similar results. Stacked bar histogram represents the percentage of cells in the four quadrants.

**Figure S6. Flow cytometric analysis of autophagy**. U266 cell line was transfected with miR-125b, miR-NC, miR-125b-Omet (Omet), miR-125b-LNA (LNA) or miR-125b-2'F (F) at a final concentration of 100 nM for 48h. After monodansylcadaverine (MDC) staining, cells were analysed on the BD Accuri C6™ flow cytometer. B and D) Replicates of the histograms reported in Fig. 4C of the main manuscript. A, C and E) Representative gating strategy of the three replicates.

**Figure S7. Flow cytometric analysis of autophagy**. U266 cell line was transfected with miR-125b, miR-NC, miR-125b-Omet (Omet), miR-125b-LNA (LNA) or miR-125b-2'F (F) at a final concentration of 100 nM for 72h. After monodansylcadaverine (MDC) staining cells were analysed on the BD Accuri C6™ flow cytometer. The histogram shows the relative intensity fluorescence intensity (MFI) with respect to control sample. The results are shown as means ± standard deviation (SD) for three independent experiments.

**Figure S8. Analysis of senescence.** Senescence-associated β-galactosidase assay. U266 cells were transfected with 100 nM of miR-125b, miR-NC, miR-125b-Omet (Omet), miR-125b-LNA (LNA) or miR-125b-2'F (F). After 72h from transfection the cells were incubated with staining solution as described in “Materials and Methods” and counted with a light microscope. The histogram shows the percent of blue cells. The results are shown as means ± standard deviation (SD) for three independent experiments.

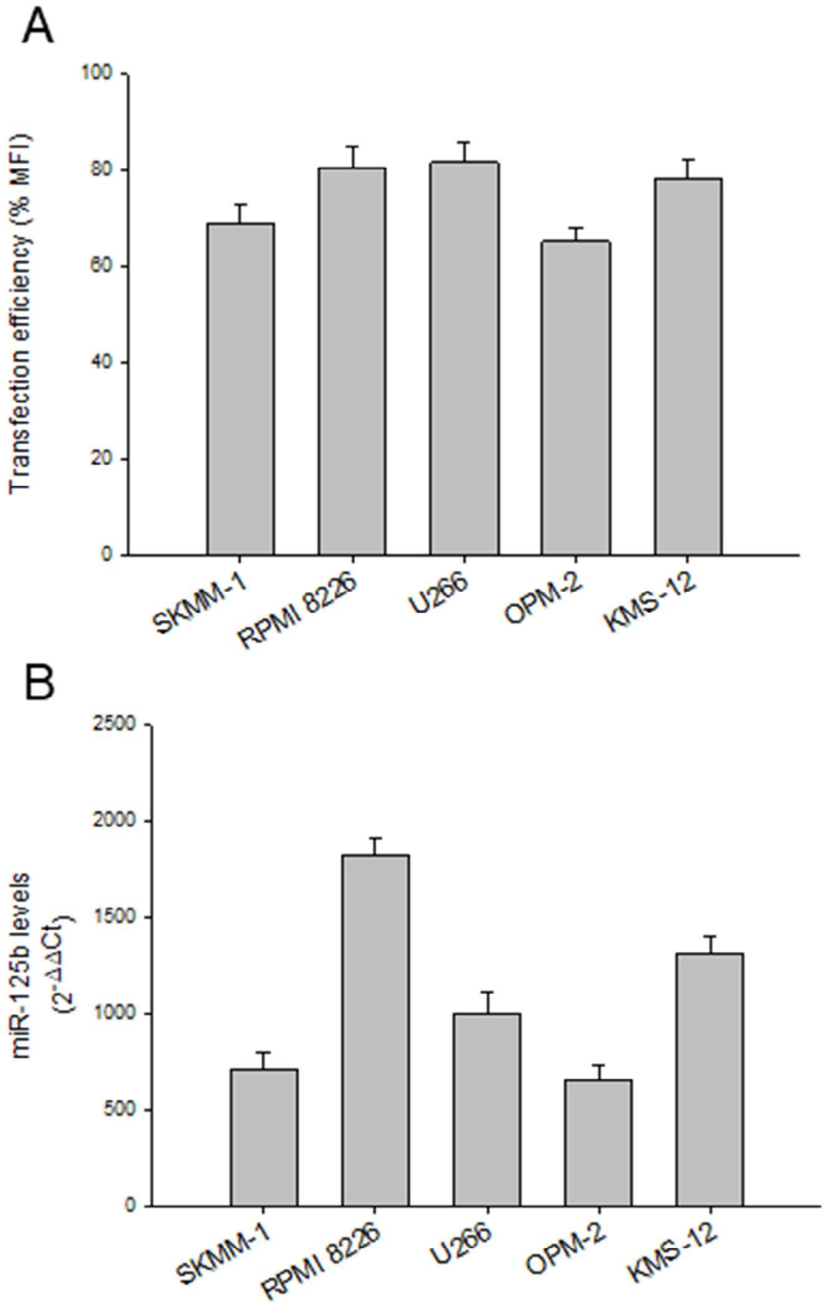
A
Transfection efficiency (% MFI)
100
80
60
40
20
0
SKMM-1
RPMI 8226
U266
OPM-2
KMS-12
B
miR-125b levels
(2-ΔΔCt)
2500
2000
1500
1000
500
0
SKMM-1
RPMI 8226
U266
OPM-2
KMS-12

**Figure S1**

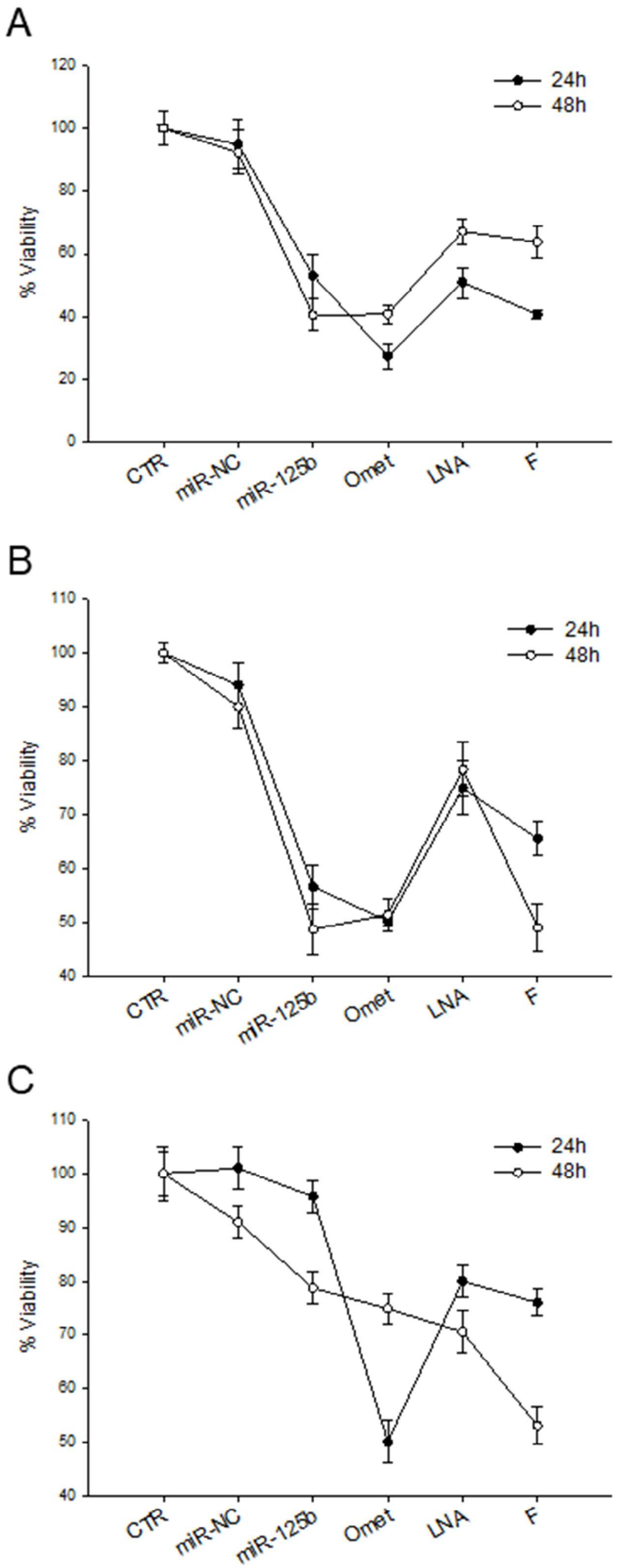
A
% Viability
0
20
40
60
80
100
120
24h
48h
CTR
miR-NC
miR-125b
Omet
LNA
F
B
% Viability
40
50
60
70
80
90
100
110
24h
48h
CTR
miR-NC
miR-125b
Omet
LNA
F
C
% Viability
40
50
60
70
80
90
100
110
24h
48h
CTR
miR-NC
miR-125b
Omet
LNA
F

**Figure S2**

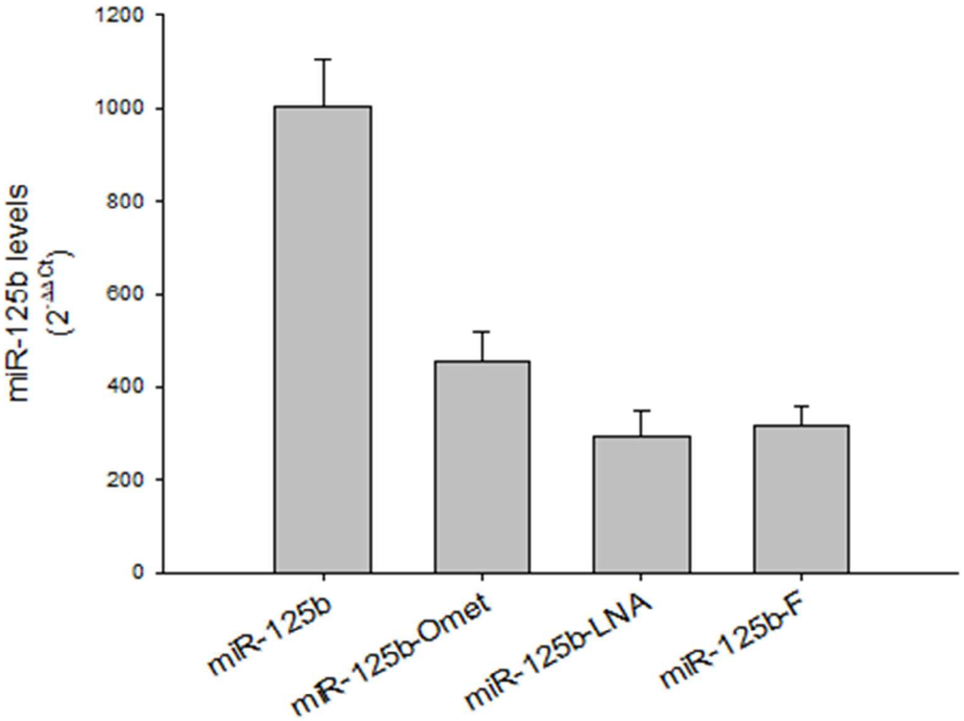

1200
1000
800
600
400
200
0
miR-125b levels
($2^{-\Delta\Delta Ct}$)
miR-125b
miR-125b-Omet
miR-125b-LNA
miR-125b-F


**Figure S3**

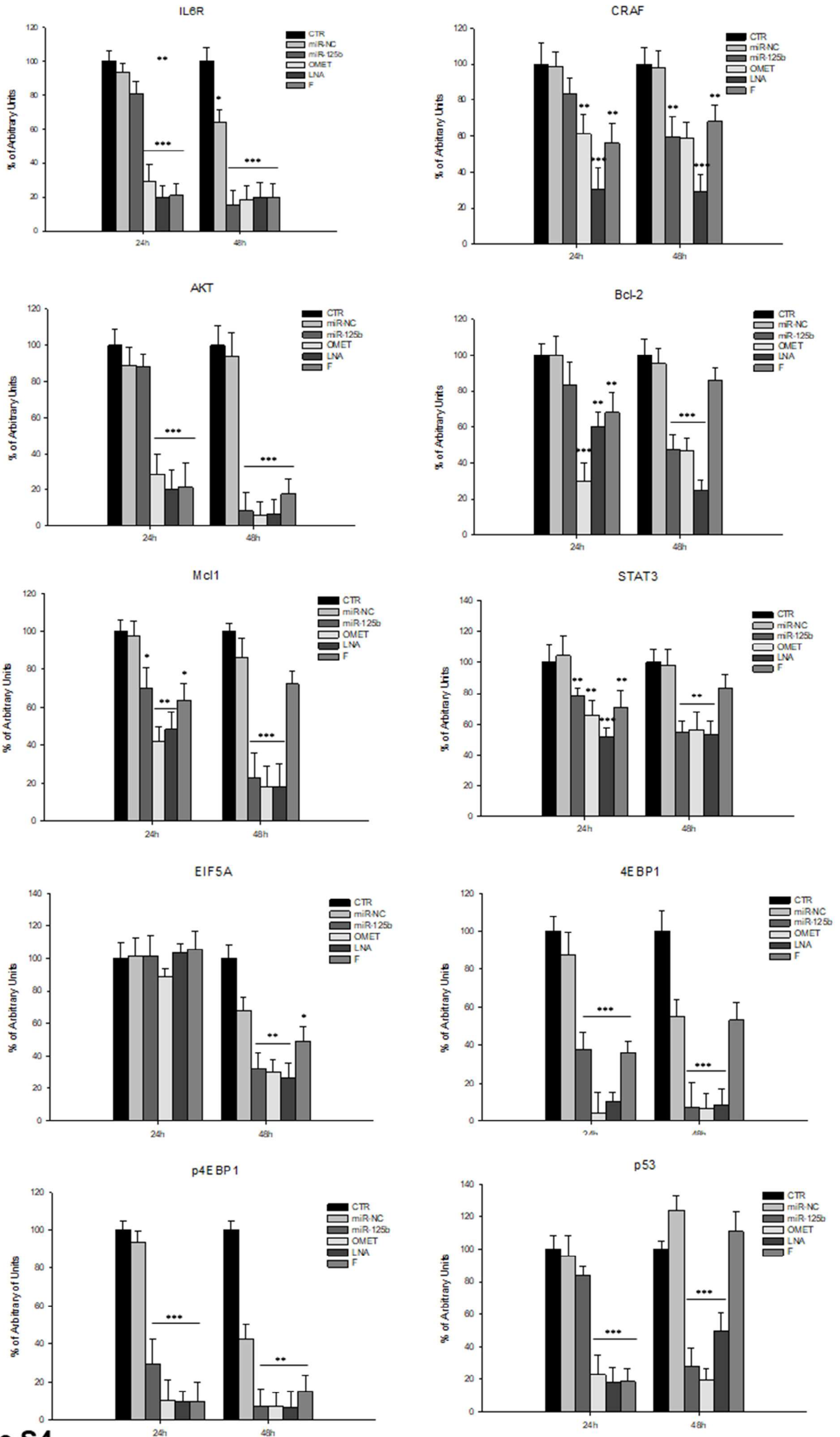
IL6R
CRAF
AKT
Bcl-2
Mcl1
STAT3
EIF5A
4EBP1
p4EBP1
p53
CTR
miR-NC
miR-125b
OMET
LNA
F
% of Arbitrary Units
% of Arbitrary of Units
24h
48h

**Figure S4**

A

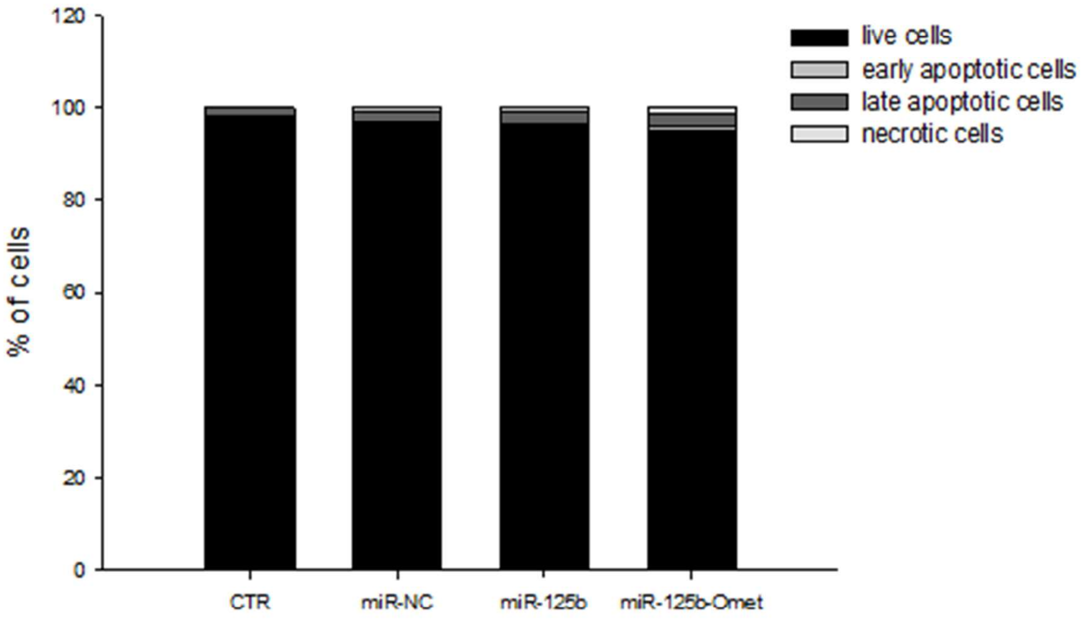
120
100
80
60
40
20
0
% of cells
live cells
early apoptotic cells
late apoptotic cells
necrotic cells
CTR
miR-NC
miR-125b
miR-125b-Omet

B

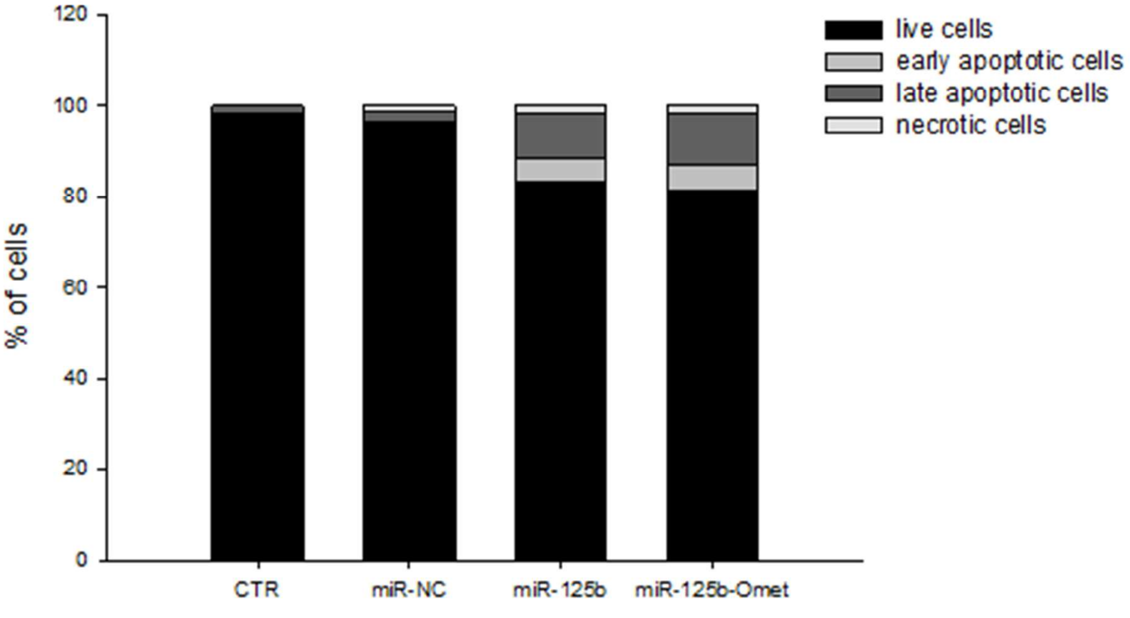
120
100
80
60
40
20
0
% of cells
live cells
early apoptotic cells
late apoptotic cells
necrotic cells
CTR
miR-NC
miR-125b
miR-125b-Omet

**Figure S5**

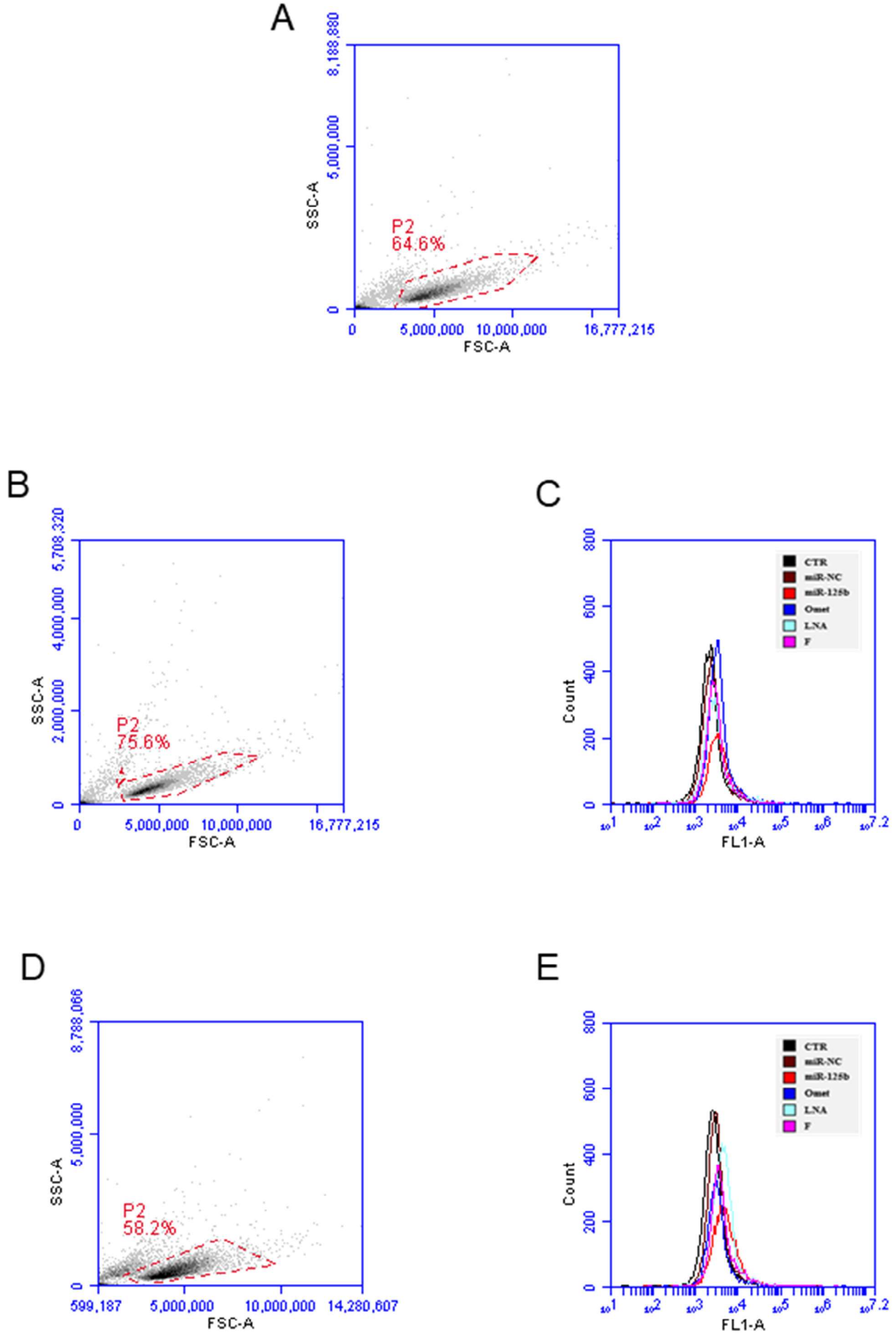
A
SSC-A
FSC-A
8,188,880
5,000,000
0
5,000,000
10,000,000
16,777,215
P2
64.6%
B
SSC-A
FSC-A
5,708,320
4,000,000
2,000,000
0
5,000,000
10,000,000
16,777,215
P2
75.6%
C
Count
FL1-A
800
600
400
200
0
CTR
miR-NC
miR-125b
Omet
LNA
F
D
SSC-A
FSC-A
8,788,066
5,000,000
0
599,187
5,000,000
10,000,000
14,280,607
P2
58.2%
E
Count
FL1-A
800
600
400
200
0
CTR
miR-NC
miR-125b
Omet
LNA
F

**Figure S6**

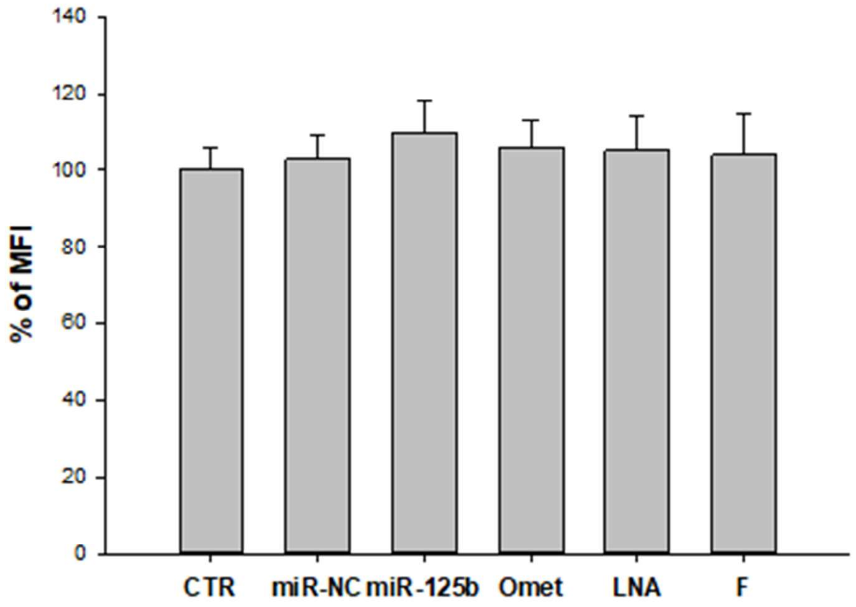


**Figure S7**

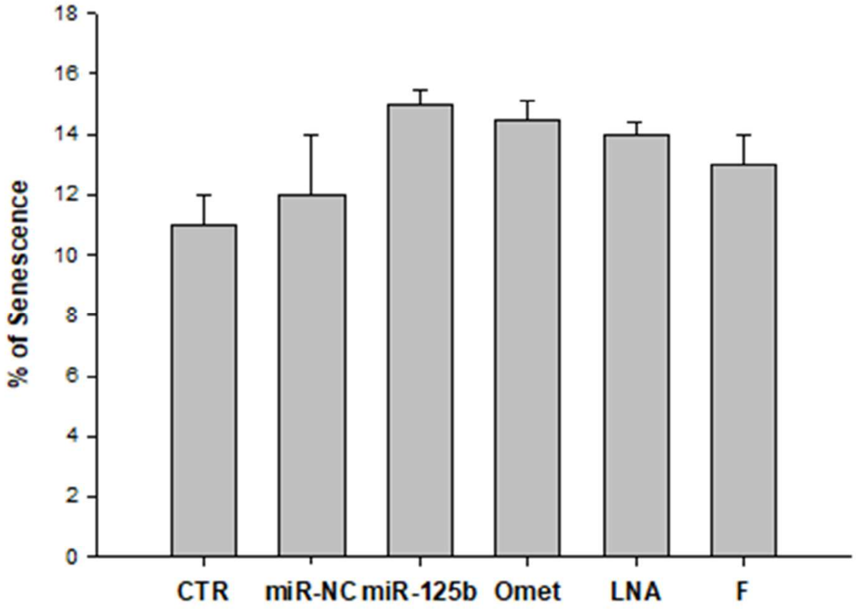
18
16
14
12
10
8
6
4
2
0
% of Senescence
CTR
miR-NC
miR-125b
Omet
LNA
F

**Figure S8**
