# Supplementary material for: miR-125b Upregulates miR-34a and Sequentially Activates Stress Adaption and Cell Death Mechanisms in Multiple Myeloma
Source: Mol Ther Nucleic Acids. 2019 Mar 13;16:391–406. doi: 10.1016/j.omtn.2019.02.023 (PMC6479071; doi:10.1016/j.omtn.2019.02.023)
Supplement: Document S2. Article plus Supplemental Information [file mmc2.pdf]

Molecular Therapy
Nucleic Acids
Original Article

miR-125b Upregulates miR-34a and Sequentially Activates Stress Adaption and Cell Death Mechanisms in Multiple Myeloma

Gabriella Misso,[1,9] Mayra Rachele Zarone,[1,9] Angela Lombardi,[1,9] Anna Grimaldi,[1] Alessia Maria Cossu,[1,2] Carmela Ferri,[1] Margherita Russo,[1] Daniela Cristina Vuoso,[1] Amalia Luce,[1] Hiromichi Kawasaki,[3] Maria Teresa Di Martino,[4] Antonella Virgilio,[5] Agostino Festa,[1] Aldo Galeone,[5] Giuseppe De Rosa,[5] Carlo Irace,[5] Massimo Donadelli,[6] Alois Necas,[7] Evzen Amler,[8] Pierosandro Tagliaferri,[4] Pierfrancesco Tassone,[4] and Michele Caraglia[1,2]

[1]Department of Precision Medicine, University of Campania "Luigi Vanvitelli," 80138 Naples, Italy; [2]IRGS, Biogem, Molecular and Precision Oncology Laboratory, Via Camporeale, 83031 Ariano Irpino, Italy; [3]Drug Discovery Laboratory, Wakunaga Pharmaceutical Co., Ltd., Hiroshima, Japan; [4]Department of Experimental and Clinical Medicine, University Magna Græcia of Catanzaro, Salvatore Venuta University Campus, 88100 Catanzaro, Italy; [5]Department of Pharmacy, School of Medicine, University of Naples Federico II, Via Domenico Montesano 49, 80131 Naples, Italy; [6]Department of Neurosciences, Biomedicine and Movement Sciences, Section of Biochemistry, University of Verona, Verona, Italy; [7]CEITEC – Central European Institute of Technology, University of Veterinary and Pharmaceutical Sciences Brno, Brno, Czech Republic; [8]Second Medical Faculty, Charles University in Prague, Prague, Czech Republic

miR-125b, ubiquitously expressed and frequently dysregulated in several tumors, has gained special interest in the field of cancer research, displaying either oncogenic or oncosuppressor potential based on tumor type. We have previously demonstrated its tumor-suppressive role in multiple myeloma (MM), but the analysis of molecular mechanisms needs additional investigation. The purpose of this study was to explore the effects of miR-125b and its chemically modified analogs in modulating cell viability and cancer-associated molecular pathways, also focusing on the functional aspects of stress adaptation (autophagy and senescence), as well as programmed cell death (apoptosis). Based on the well-known low microRNA (miRNA) stability in therapeutic application, we designed chemically modified miR-125b mimics, laying the bases for their subsequent investigation in *in vivo* models. Our study clearly confirmed an oncosuppressive function depending on the repression of multiple targets, and it allowed the identification, for the first time, of miR-125b-dependent miR-34a stimulation as a possible consequence of the inhibitory role on the interleukin-6 receptor (IL-6R)/signal transducer and activator of transcription 3 (STAT3)/miR-34a feedback loop. Moreover, we identified a pattern of miR-125b-co-regulated miRNAs, shedding light on possible new players of anti-MM activity. Finally, functional studies also revealed a sequential activation of senescence, autophagy, and apoptosis, thus indicating, for the first two processes, an early cytoprotective and inhibitory role from apoptosis activation.

INTRODUCTION

Over the last decade, the biology of multiple myeloma (MM) has been extensively studied to define the complex interplay between the signaling pathways accountable for its intricate physiopathology. MM is supported by the bone marrow microenvironment (BMM), since it can promote myeloma growth and survival, and it is also responsible for bone damage, the most common clinical feature in MM patients.[1] It is noteworthy that more than 80%–90% of MM patients develop bone involvement;[2] other hallmarks include hypercalcemia, renal dysfunction, and anemia.[2] The earliest phase before the development of symptomatic MM is termed monoclonal gammopathy of undetermined significance (MGUS).[3] Another asymptomatic phase following MGUS is smoldering MM (SMM), characterized by a 10% average risk of progression to myeloma per year for the first 5 years from the occurrence of the disease, decreasing to 3% per year for the next 5 years and to 1%–2% per year for the next 10 years.[4] When pre-malignant disorders evolve into MM, the overt symptoms arise with clinically relevant end-organ damage related to the plasma cell proliferative disorder.[5]

The final phase is plasma cell leukemia (PCL), an aggressive disease endpoint characterized by the existence of extramedullary clones

Received 21 September 2018; accepted 26 February 2019;
https://doi.org/10.1016/j.omtn.2019.02.023.

[9]These authors contributed equally to this work.

Correspondence: Michele Caraglia, Department of Precision Medicine, University of Campania "Luigi Vanvitelli," 80138 Naples, Italy.
E-mail: michele.caraglia@unicampania.it
Correspondence: Gabriella Misso, Department of Precision Medicine, University of Campania "Luigi Vanvitelli," 80138 Naples, Italy.
E-mail: gabriella.misso@unicampania.it
Correspondence: Maria Teresa Di Martino, Medical Oncology Unit, Department of Experimental and Clinical Medicine, University Magna Græcia of Catanzaro, Salvatore Venuta University Campus, 88100 Catanzaro, Italy.
E-mail: teresadm@unicz.it

This is an open access article under the CC BY license (http://creativecommons.org/licenses/by/4.0/).

and rapid progression to death.[5] In detail, bone disease occurs when plasma cells directly interact with the BMM, which results in an increased rate of osteoclast-mediated bone resorption and decreased osteoblast-mediated bone formation.[2] This imbalance is caused by the dysregulation of several cytokines, chemokines, growth factors, and other components secreted by bone marrow stromal cells (BMSCs), which contribute to a symbiotic cycle that maintains an MM-promoting microenvironment and allows the tumor cells to secrete interleukin-1β (IL-1β), tumor necrosis factor alpha (TNF-α), transforming growth factor β (TGF-β), VEGF, and interleukin-6 (IL-6).[6] Binding of IL-6 to the IL-6 receptor (IL-6R) triggers the activation of at least three signaling pathways: the Janus kinase (JAK)/signal transducer and activator of transcription (STAT) pathway, the Ras/mitogen-activated protein kinase (MAPK) pathway, and the PI3K/Akt pathway.[7,8]

The identification of the key role exerted by the BMM in support of MM cell growth and survival has opened the way to innovative target-based therapeutic approaches. In this context, microRNAs (miRNAs) are promising tools not only for the early detection but also for the treatment of MM. miRNAs act either as oncogenes (oncomiR) or tumor suppressors, regulating simultaneously the expression of multiple target genes.[9–11] The main challenge for an effective miRNA-based therapy still includes the effective delivery of the appropriate miRNA to the BMM and its uptake by malignant plasma cells in the absence of off-target effects.[12] Potential strategies based on miRNA therapeutics basically rely on miRNA inhibition or miRNA replacement approaches and take benefit respectively from the use of synthetic miRNAs[13–17] or specific miRNA inhibitors.[18–20] Among the miRNAs dysregulated in MM, not much is known about miR-125b function, except the reported inactivation of p53,[21,22] suggestive of an oncogenic role. However, divergent experimental evidence has demonstrated the tumor-suppressive function of miR-125b in the U266 MM cell line, since its transduction was able to induce a significant increase in death rate compared to control cells by the downregulation of BLIMP-1, IRF4, and SYNDECAN-1 (CD138), a cell surface molecule expressed by myeloma and primary plasma cells.[23] We have previously demonstrated that miR-125b was deregulated in TC2/3 molecular subtypes of MM. Moreover, anti-myeloma activity was achieved via direct downregulation of IRF4 and its downstream effector BLIMP-1. All together these findings led us to conclude that miR-125b has tumor suppressor activity and offers a new means for miRNA replacement strategies.[24]

The need to generate additional information into the biology of miR-125b in the translational perspective has prompted us to carry out the present study. The basic premise of our work was the identification, in TargetScan (http://www.targetscan.org/vert_72/), of a series of appealing predicted miR-125b targets; among them we recognized the main promoter and effector factors responsible for maintaining the myeloma microenvironment and for conferring resistance to conventional therapies. Moreover, functional studies have also proven useful in identifying a temporal correlation in the activation of autophagy, senescence, and apoptosis. This cross-regulation has its roots in a series of studies showing that, in senescent cells, autophagy serves as a mechanism of adaptation to stress; in fact, autophagosomes have been shown to accumulate in senescent fibroblasts to facilitate the renewal of cytosolic compounds and organelles.[25,26] Otherwise, apoptosis resistance of both autophagic and senescent cells is well documented in the literature.[27–29]

In detail, here we have analyzed the antiproliferative *in vitro* activity promoted by miR-125b and its synthetic analogs, correlating it with the p53 mutational status and with the expression of several targets with regulatory function on multiple intracellular pathways activated by growth stimuli. We have exploited a series of chemical modifications (2′-O-Methylation [2′-Omet], 2′-Fluorination [2′-F] or locked nucleic acid [LNA]) aimed at both improving the resistance to nucleases and increasing the stability and binding specificity of the mRNA-miRNA duplex.[30,31] Our experimental results have allowed us to identify the best chemical modifications in terms of anti-myeloma activity, laying the bases for a subsequent use of such compounds in *in vivo* models to assess the actual biological stability. Moreover, we have shed light on the co-regulation of multiple miRNAs, performing miRNome-wide expression profiling. Thereafter, we validated the effects of miR-125b, as well as of its modified analogs, in modulating the expression of the tumor suppressor miR-34a, identifying, for the first time, a regulatory loop between these two miRNAs. Finally, based on the current knowledge that describes senescence as a process that can trigger autophagy as a mechanism of adaptation to stress[25–32] and, at the same time, as a process that reduces cell reactivity to apoptotic stimuli,[33] functional studies were performed to analyze the effect of miR-125b ectopic expression on the modulation of both stress adaptation (autophagy and senescence) and programmed cell death (apoptosis) in MM cells, identifying a sequential activation of these processes.

RESULTS

Mutational Analysis of MM Cells

The identification of common and rare genomic variants in candidate regions of the human genome is essential to better understand the complex human disease etiology. Mutational analysis of U266, SKMM-1, and RPMI 8226 MM cell lines was performed as described in the Materials and Methods. Genetic profiling of the MM cell lines has highlighted deleterious mutations in several genes involved in cell proliferation and differentiation processes. Next-generation sequencing (NGS) was performed on the Ion Torrent PGM, using a panel that contains amplicons to detect currently known cancer-associated mutations in tumor driver genes. Data obtained showed that U266 cells are mutated in MET, TP53, and BRAF genes; SKMM-1 cells are mutated in CSDE1 (NRAS upstream gene), PTEN, and TP53; RPMI 8226 cells are mutated in a greater number of mutated genes, in particular ERBB4, PIK3CA, EGFR, KRAS, and TP53. The results of molecular investigations are summarized in Table S1.

All three lines showed single-nucleotide variants (SNVs) in the TP53 gene, but they are different from one another. Furthermore, three new

mutations, designated as novel, have been found. Somatic mutations in the TP53 gene are one of the most frequent alterations in human cancers, and the diverse types and positions may inform on the nature of mutagenic mechanisms involved in cancer etiology. To clarify the clinical and functional impacts of these variants, a literature search was done using the principal TP53 variants database IARC TP53 Database (R18 version)[34] (Table S2). Two mutants (p.R175G in SKMM-1 and p.E285K in RPMI 8226) showed a complete loss of transactivation activities, one mutant (p.A161T in U266) was partially functional, and two mutants (p.A161fs in U266 and p.P72R in SKMM-1) showed an unknown effect.

miR-125b Expression in MM Cell Lines

To select an MM cell system suitable for the study of biological effects induced by miR-125b replacement, we analyzed, by qRT-PCR, basal miR-125b expression in a panel of five MM cell lines (RPMI 8266, KMS-12, OPM-2, SKMM-1, and U266). We observed that RPMI 8266 and KMS-12 cells had significantly higher miR-125b levels compared to OPM-2, SKMM-1, and U266 cell lines. In detail, miR-125b expression was, respectively, 20- and 45-fold higher in KMS-12 and RPMI 8226 cell lines, if compared to U266 cells (Figure 1A). Moreover, no significant differences in miR-125b levels were observed among U266, OPM-2, and SKMM-1 cell lines.

Design of Chemically Modified Oligonucleotides

Improvement of miRNA stability is a key prerequisite to perform an effective replacement strategy. Therefore, we exploited a series of chemical modifications at the 2′ position of ribose (Figure 1B) to increase both the nucleases' resistance in the biological environment and the stability and/or binding specificity of the miRNA-mRNA duplex.[30] It is well known that most of RNA degradation mechanisms catalyzed by nucleases involve the 2′-OH of the ribose moiety; therefore, chemical modifications at this site produce a considerable stabilization. Moreover, it is noteworthy that the introduction of a chemical moiety in the 2′ position favors the typical double-helix conformation of the miRNA-mRNA duplex.[30,31] Given these assumptions, we introduced the following chemical modifications in the sugar moiety of miR-125b: 2′-Omet, 2′-F, and LNA. LNA comprises a class of bicyclic RNA analogs in which the furanose ring in the sugar-phosphate backbone is chemically locked in an RNA mimicking N-type (C3′ endo) conformation by the introduction of a 2′-O, 4′-C methylene bridge, which favors RNA A-type duplex geometry. This modification significantly increases Tm (melting temperature) and is also very resistant to nuclease. Due to the large increase in Tm conferred by LNAs, they also increase primer dimer as well as self-hairpin formation. These modified oligonucleotides, along with unmodified miR-125b mimic, were subsequently tested for miRNA replacement in our MM cellular models.

miR-125b Ectopic Expression in MM Cells Affects Cell Viability

Based on qRT-PCR data and on the different transfection efficiencies showed by the MM cell lines (Figures S1A and S1B), we selected SKMM-1 and U266 cells exhibiting low miR-125b levels and RPMI 8226 cells expressing the highest miRNA levels. These cell lines were transiently transfected by electroporation either with miRNA scramble (miR-NC), used as a negative control, or with synthetic miR-125b mimic or its modified analogs. miRNAs were transfected at a final concentration of 100 nM that was selected on the basis of previous standardization procedures. The subsequent effects on cell viability and proliferation were evaluated at 24 and 48 h from transfection by trypan blue exclusion dye (Figures 1C and 1H) and by MTS assay (Figures S2A–S2C).

We observed that miR-125b replacement in the U266 cell line (Figures 1C and 1D; Figure S2A) induced, already after 24 h, a significant decrease in cell viability. In detail, transfection with miR-125b or miR-125b-LNA caused a 40% reduction in cell viability compared to control (CTR, untreated cells); a further decrease was observed with miR-125b-2′F transfection, but the highest viability inhibition (more than 70%) was induced by miR-125b-Omet. This inhibitory effect was even more pronounced 48 h after electroporation.

Analysis of viability in the SKMM-1 cell line (Figures 1E and 1F; Figure S2B), performed under the same experimental conditions, also showed significant perturbation, consisting of a 60% reduction compared to CTR, at both 24 and 48 h after electroporation with miR-125b. Similar results were obtained when cells were transfected with miR-125b-Omet, whereas miR-125b-2′F induced a slightly lower inhibition after 24 h and a powerful inhibitory effect at 48 h. miR-125b-LNA induced a less pronounced cell growth inhibition.

Finally, for RPMI 8226, a weak decrease in cell viability was observed after replacement with miR-125b, although a more pronounced antiproliferative effect was achieved by chemically modified miRNAs. In detail, miR-125b-2′F induced the most significant cell viability reduction (about 50%) at 24 h after electroporation (Figures 1G and 1H; Figure S2C). At 48 h, the percentage of viability inhibition was between 20% and 25% for miR-125b, miR-125b-LNA, and miR-125b-2′F transfections and up to 50% for miR-125b-Omet, respectively.

These data demonstrate that, for the analyzed MM cell lines, we can establish a correlation between basal miR-125b levels and the effect on viability induced by miRNA replacement. In fact, RPMI 8226 cells, which express high basal miR-125b levels, were not affected by enforced miRNA expression. Moreover, among the modified analogs, miR-125b-Omet appeared to be the most effective in inducing antiproliferative effects.

miRNA Profiling in miR-125b-Transfected Cells

On the basis of qRT-PCR data, as well as on cell responsiveness to replacement, we selected the U266 cell line to study, by qRT-PCR array cards, the modulation of a set of highly characterized miRNAs (Figure 2A). Of the 384 tested miRNAs, excluding the 3 endogenous controls and the 6 negative controls, 161 were expressed in the analyzed populations. Stringent criteria were used to identify differentially expressed miRNAs with a C_t value above 10 and below 32. According to this, more than 2-fold altered expression compared to

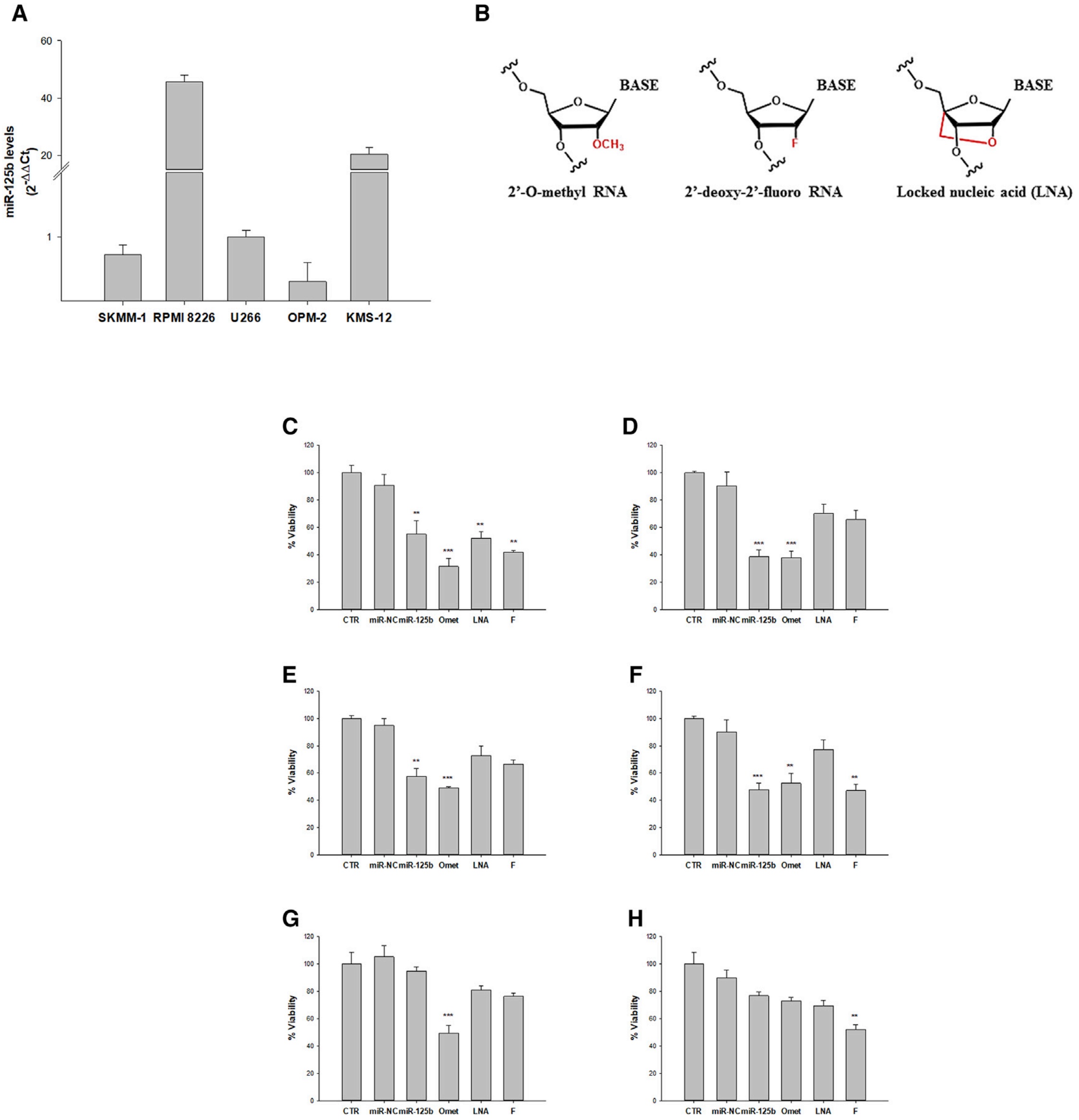

Figure 1. miR-125b Levels in MM Cell Lines and Cell Viability Modulation after miR-125b Mimic and Its Modified Analog Transfection

(A) Analysis of basal miR-125b levels by qRT-PCR in SKMM-1, RPMI 8226, U266, OPM-2, and KMS-12 cell lines. Each experiment was repeated at least three times and data are shown as mean ± SD. (B) Schematic representation of chemical modifications: 2′-O-Methylation, 2′-Fluorination, and locked nucleic acid (LNA) were introduced in the sugar moiety of miR-125b mimics. (C–H) Trypan blue cell assay of MM cell lines transfected with 100 nM miR-125b, miR-NC, miR-125b-Omet (Omet), miR-125b-LNA (LNA), or miR-125b-2′F (F). MM cell lines U266 (C and D), SKMM-1 (E and F), and RPMI 8226 (G and H) were analyzed after 24 h (C, E, and G) and 48 h (D, F, and H) from transfection. CTR, untreated cells used as control. Each experiment was repeated at least three times and the data are shown as a mean ± SD. *p $\leq$ 0.05, **p $\leq$ 0.01, ***p $\leq$ 0.001.

control (miR-NC-transfected cells) was observed for 36 miRNAs (excluding the same miR-125b). Among these, 28 were upregulated and 8 downregulated. In detail, within the upregulated miRNAs, 23 were expressed between 2- and 3-fold more than control, 4 between 3- and 5-fold, and 1 over 5-fold. Within the downregulated miRNAs, 3 were expressed between 2- and 3-fold less than control and 5 between 3- and 5-fold less (Table 1).

We focused our attention on the 11 up- or downregulated more than 3-fold (Figure 2B). All these miRNAs have been already characterized for their role in cancer, but not all of them have already been defined for a specific function in MM. Let-7c, encoded at the same cluster (21q21) of miR-125b, plays an oncosuppressive role in several cancers; its expression is reduced in both tissue and sera of breast cancer patients, hypothesizing for this miRNA a role as novel and valuable

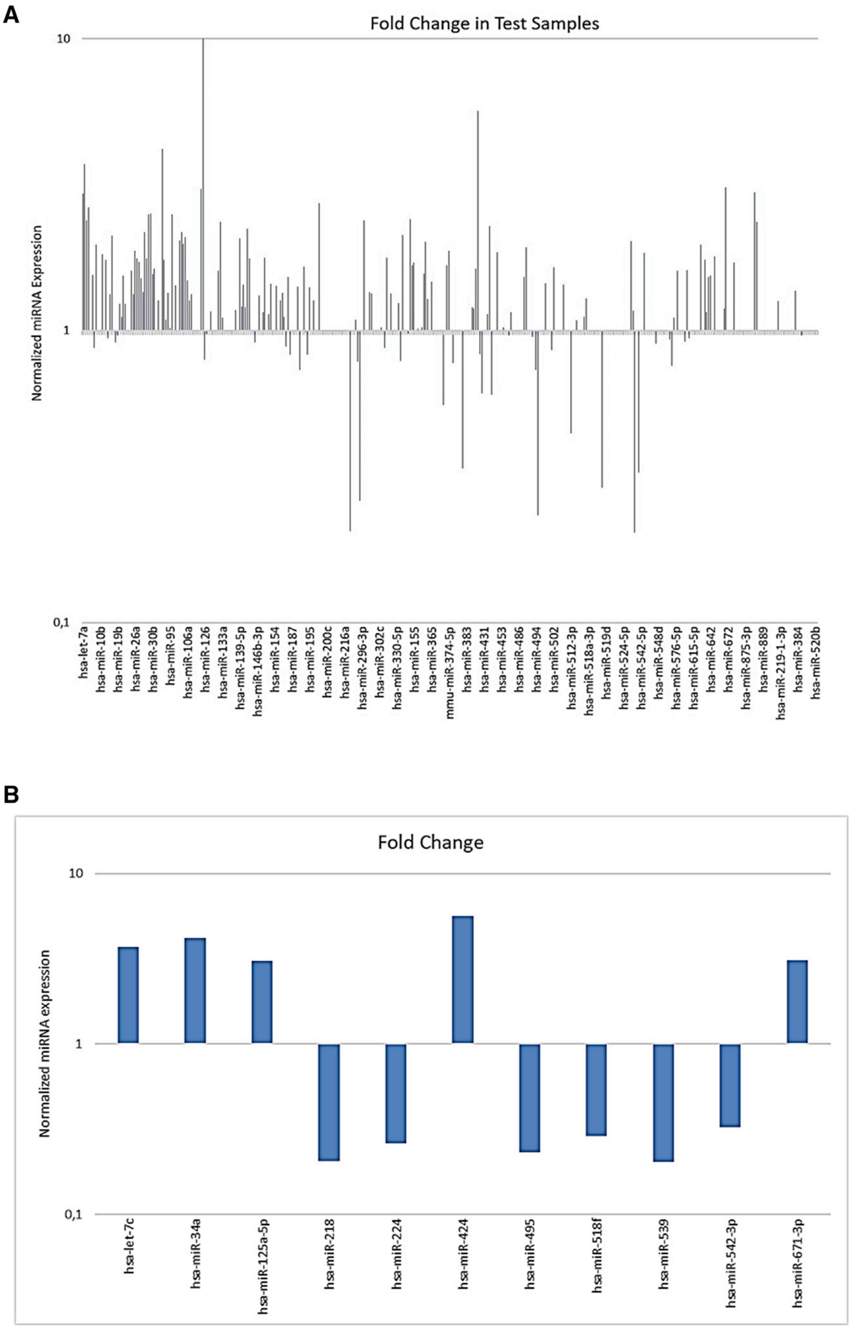

Figure 2. miRNA Profile in U266 Cells Overexpressing miR-125b versus NC-Transfected Cells

(A) Analysis of differentially expressed miRNAs between U266 cells transfected with 100 nM miR-125b or miR-NC for 48 h. Histogram shows all up- and downregulated miRNAs whose Ct values are between 10 and 32 and their corresponding fold change. (B) Expression levels of the significant up- and downregulated miRNAs after 100 nM miR-125b or miR-NC transfection for 48 h in U266 cells. Fold changes were relative to miR-125b-transfected cells matched with control (NC-transfected) cells. Only miRNA expression fold changes >3 are presented in this graph.

diagnostic biomarker.[35] Let-7c expression is also downregulated in castration-resistant prostate cancer cells, where its ectopic expression induced growth arrest and, in parallel, its suppression enhanced the ability of androgen-sensitive prostate cancer cells to grow in androgen-deprived conditions.[36] An oncosuppressive role was recently observed also in cholangiocarcinoma.[37] The member of the miR-125b seed family, miR-125a-5p, that induces oncosuppression and chemosensitization, respectively, in non-small-cell lung and breast cancers,[38,39] was also upregulated after miR-125b replacement. A cancer-related tumor-suppressive (glioma and ovarian cancer[40,41]) or tumor-promoting (gastric and hepatocellular cancers[42,43]) effect was also registered in previous studies for another significantly upregulated miRNA, miR-424. Among the downregulated miRNAs, a group of evidence demonstrated that miR-218 plays pivotal roles in tumorigenesis by targeting many oncogenes related to proliferation, apoptosis, and invasion.[44] Also miR-224 has a well-known role in promoting migration and invasion,[45] and it contributes to the malignant progression of lung cancer, meningioma, and hepatocellular carcinoma.[46–48] miR-495 induces cell proliferation, invasion, and migration in both gastric[49] and bladder cancers,[50] but an opposite effect was observed in colorectal cancer.[51] Both miR-539 and miR-542-3p have been shown to have an oncosuppressive function in several cancers,[52–55] although their role is still poorly explored and, specifically, their function in MM has not been investigated. Even less defined is the role of miR-518f, which was found to be, together with 28 other miRNAs, significantly downregulated in an *in vitro*-selected highly invasive endometrial cancer cell line.[56] Among the most upregulated miRNAs, we found the well-known oncosuppressor miR-34a, which contributes to p53 downstream effects on proliferation arrest and apoptosis induction, by targeting c-MYC, CDK6, and c-MET.[9] We have previously demonstrated its downregulation in MM, where, in addition, its ectopic expression was able to down-modulate both Erk and Akt pathways and also to induce apoptosis through pro-caspase-6 and -3 cleavage.[14]

Our results from miRNome analysis offer for the first time an interesting overview of miRNA co-regulation in MM after miR-125b replacement, and they reveal the involvement of a number of miRNAs whose roles had been hitherto unexplored in this neoplasm.

Table 1. Upregulated and Downregulated miRNAs in miR-125b-Transfected Cells Compared to miR-NC-Transfected Cells

miRNA	Fold Change	Modulation
hsa-let-7a	2.95	upregulated
hsa-let-7c	3.72	upregulated
hsa-let-7d	2.38	upregulated
hsa-let-7e	2.65	upregulated
hsa-miR-18a	2.12	upregulated
hsa-miR-28	2.18	upregulated
hsa-miR-29b	2.51	upregulated
hsa-miR-29c	2.51	upregulated
hsa-miR-34a	4.20	upregulated
hsa-miR-96	2.51	upregulated
hsa-miR-100	2.035	upregulated
hsa-miR-101	2.17	upregulated
hsa-miR-105	2.09	upregulated
hsa-miR-125a-5p	3.07	upregulated
hsa-miR-132	2.36	upregulated
hsa-miR-139-5p	2.07	upregulated
hsa-miR-142-3p	2.24	upregulated
hsa-miR-199a-3p	2.72	upregulated
hsa-miR-218	4.86	downregulated
hsa-miR-224	3.84	downregulated
hsa-miR-296	2.38	upregulated
hsa-miR-335	2.13	upregulated
hsa-miR-339-5p	2.42	upregulated
hsa-miR-362-3p	2.02	upregulated
hsa-miR-381	2.97	downregulated
hsa-miR-424	5.66	upregulated
hsa-miR-449b	2.29	upregulated
hsa-miR-495	4.48	downregulated
hsa-miR-512-3p	2.24	downregulated
hsa-miR-518f	3.45	downregulated
hsa-miR-532-3p	2.02	upregulated
hsa-miR-539	4.92	downregulated
hsa-miR-542-3p	3.06	downregulated
hsa-miR-671-3p	3.10	upregulated
hsa-miR-886-3p	2.97	upregulated
hsa-miR-886-5p	2.36	upregulated

miR-34a Expression in MM Cell Lines

Taking into account the ubiquitous oncosuppressive role of miR-34a and, more specifically, also the widely investigated molecular mechanisms mediating its anti-MM activity, already reported in our previous papers,[13,14,16,17] we have validated the actual stimulatory effect of miR-125b on miR-34a expression. First, we analyzed basal miR-34a levels in all MM cell lines by qRT-PCR. We found that, as for miR-125b, also miR-34a was largely expressed in the RPMI 8226 cell line, while U266 and OPM-2 expressed lower and similar basal levels of miR-34a, whose expression exceeded, in turn, 2.8-fold and 8.2-fold, respectively, the expression levels in KMS-12-PE and SKMM-1 cells (placing U266 miR-34a levels as unitary value) (Figure 3A). Thereafter, we performed a qRT-PCR on the U266 cell line at 24 h from transfection with or without miR-NC or miR-125b (Figure 3B). Supporting our preliminary microarray data, miR-125b replacement induced an almost 4-fold upregulation of miR-34a.

Hence, a putative miR-125b-mediated tumor-suppressive role in our cell model could be due, at least in part, to the increase in miR-34a expression.

Modulation of miR-125b Targets in the U266 Cell Line

U266 cells transfected with miR-NC or miR-125b wild-type were processed for the analysis of the main tumor promoter and tumor-suppressive miR-125b targets identified by the TargetScan platform and recent literature (Table 2). In detail, cells electroporated either with miR-125b or its modified analogs (see Figure S3 for transfection efficiency) were processed for western blot analysis of several molecular mediators of proliferation, survival, and cell death processes (Figures 3C and 3D; Figure S4). We observed that, 24 h after transfection, miR-125b induced a significant reduction in both expression and activity of the negative protein translational regulator 4EBP1, reaching a total absence of signal 24 h later. Furthermore, it was observed, for the same protein, an enhancement of the inhibitory effect when cells were electroporated with miR-125b-Omet or miR-125b-LNA. 4EBP1 phosphorylation seemed to follow the expression levels of the corresponding total protein.

Interestingly, at the first time point, IL-6R, C-Raf, Akt, Bcl-2, and Mcl-1 were slightly downregulated by miR-125b transfection; however, their expression levels were significantly reduced or completely inhibited following miR-125b-Omet or miR-125b-LNA transfection. This inhibitory effect was more significant 48 h after transfection. Moreover, the oncogenic transcription factor STAT3 was significantly reduced already 24 h after miR-125b-Omet transfection, and it was even further inhibited at 48 h; this effect was also found in cells transfected with miR-125b and, partly, in cells transfected with miR-125b-LNA. The targeting efficiency of both miR-125b and its modified analogs was also confirmed for EIF5A, particularly at 48 h. Transfection with the chemically modified miR-125b analogs showed an inhibitory effect also on p53 expression at 24 h, if compared with the wild-type miRNA that showed a more significant effect at 48 h. This specific modulation, which should lead to a global oncogenic effect, in this cellular model where p53 mutation is associated with loss of function, was not able to affect the p53-regulated network.

The observed miR-125b-mediated inhibition of STAT3 could be one of the putative mechanisms underlying the upregulation of miR-34a expression induced by miR-125b replacement. In fact, it is already known that STAT3 downregulates miR-34a, and, therefore, miR-125b could be involved in the feedback loop IL-6R/STAT3/miR-34a described by Rokavec et al.[57] This mechanism, as well as the

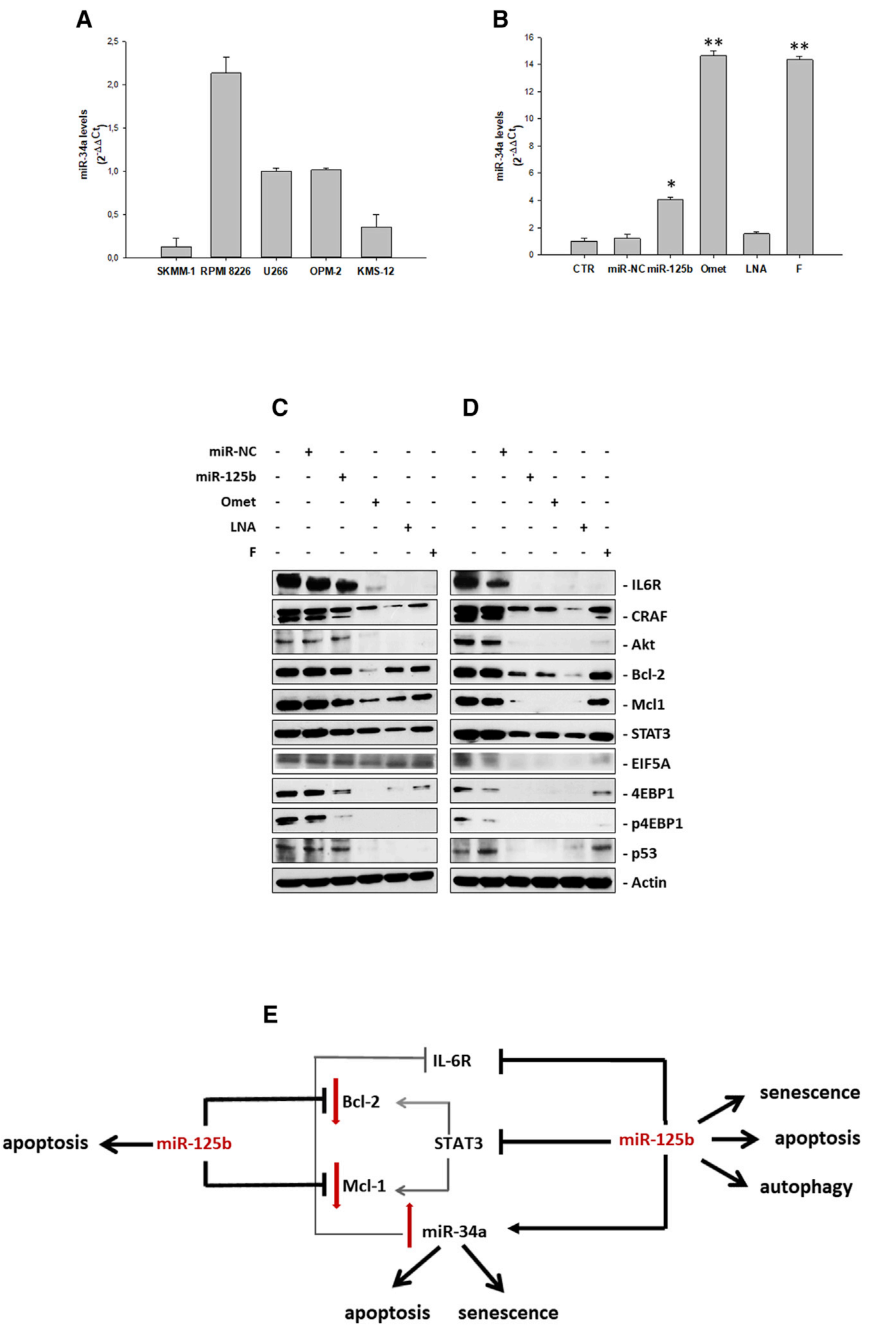

Figure 3. Modulation of Both miR-125b Targets and miR-34a Expression in MM Cells after Ectopic Expression of miR-125b or Its Modified Analogs

(A) Analysis of basal miR-34a levels by qRT-PCR in SKMM-1, RPMI 8226, U266, OPM-2, and KMS-12 cell lines. Each experiment was repeated at least three times and data are shown as mean ± SD. (B) miR-34a levels in U266 MM cell lines after ectopic expression of miR-125b mimics or its modified analogs. Analysis of miR-34a levels by qRT-PCR is shown in the U266 cell line after 48 h from transfection with 100 nM miR-125b, miR-NC, miR-125b-Omet (Omet), miR-125b-LNA (LNA), or miR-125b-2′F (F). Each experiment was repeated at least three times and data are shown as mean ± SD. $*p \leq 0.05$, $**p \leq 0.01$. (C and D) Western blot analysis of U266 cells transfected with 100 nM miR-125b, miR-NC, miR-125b-Omet (Omet), miR-125b-LNA (LNA), or miR-125b-2′F (F). After 24 h (C) and 48 h (D) from transfection, the cells were collected for western blotting analysis. Subsequently, the expressions of IL6-R, cRaf, Akt, Bcl-2, Mcl1, STAT3, pSTAT3, EIF5A, 4EBP1, p4EBP1, and p53 were evaluated after blotting with specific antibodies, as described in the Materials and Methods. The actin protein was used as a loading control. (E) Scheme representing the molecular mechanism of miR-125b-induced oncosuppressive effect. Bcl-2, Mcl-1, IL-6R, and STAT3 are miR-125b validated targets, as we have herein demonstrated. STAT3 has a great impact in transcriptional activation of pro-survival mediators Bcl-2 and Mcl-1 and, in turn, Bcl-2 can induce STAT3 activation.[7,89] STAT3 silencing by miR-125b inhibits Bcl-2 and Mcl-1 expressions, already repressed by miR-125b itself. In addition, the miR-125b-mediated simultaneous inhibition of IL-6R and STAT3 induces a clear upregulation of the tumor suppressor miR-34a, which, in turn, can further down-modulate IL-6R expression.

interference of miR-125b with other targets involved in the regulation of apoptosis, autophagy, and senescence, have been described in Figure 3E.

Overall, the target's silencing induced by miR-125b-Omet was observed earlier than miR-125b-LNA and miR-125b-2′F, while a slight effect was observed 24 h after not-modified-miR-125b transfection. Moreover, in most cases, the effect on target protein expression was lost or strongly reduced at 48 h in cells transfected with miR-125b-2′F.

These data suggest that miR-125b transfection and, more significantly, miR-125b-Omet and miR-125b-LNA transfections, induce a considerable perturbation of the major mediators of cell death and proliferation processes in the U266 cell line.

Cell Death Processes Induced by miR-125b Replacement

To investigate the functional role of miR-125b, we studied the possible induction of apoptosis and/or autophagy in the U266 cell line. In detail, cells transfected without or with miR-NC, miR-125b, miR-125b-Omet, miR-125b-LNA, and miR-125b-2′F were stained with Annexin V and propidium iodide (PI) or with Monodansylcadaverine (MDC) and processed for flow cytometry at 24, 48, and 72 h from transfection. The results showed that both miR-125b and miR-125b-Omet transfection induced a significant increase in late apoptotic cells after 72 h (Figures 4A and 4B), although no evidence of apoptosis was observed in previous times (Figure S5). On the other hand, the same treatments

Table 2. Predicted miR-125b Targets

<table>
<tr><th></th><th>Predicted consequential pairing of target region (top) and miRNA (bottom)</th><th>Site type</th><th>Context++ score</th><th>Context++ score percentile</th><th>Weighted context++ score</th><th>Conserved branch length</th><th>P_{CT}</th><th>Source</th></tr>
<tr><td>Position 836-842 of IL6R 3' UTR
hsa-miR-125b-5p</td><td>5' ...UGAAUAAUACAGUAUCUCAGGGC...
| | | | | | |
3' AGUGUUCAAUCCCAGAGUCCCU</td><td>7mer-m8</td><td>-0.28</td><td>94</td><td>-0.28</td><td>4.356</td><td>0.73</td><td>Targetscan</td></tr>
<tr><td>Position 641-648 of RAF1 3' UTR
hsa-miR-125b-5p</td><td>5' ...CCGCAUCUCAGCCCUCUCAGGGA...
| | | | | | |
3' AGUGUUCAAUCCCAGAGUCCCU</td><td>8mer</td><td>-0.33</td><td>96</td><td>-0.33</td><td>2.297</td><td>< 0.1</td><td>Targetscan</td></tr>
<tr><td>Position 2419-2426 of BCL2 3' UTR
hsa-miR-125b-5p</td><td>5' ...ACCUCCCCGGCGGGCCUCAGGGA...
| | | | | | |
3' AGUGUUCAAUCCCAGAGUCCCU</td><td>8mer</td><td>-0.13</td><td>78</td><td>-0.12</td><td>3.753</td><td>0.82</td><td>Targetscan</td></tr>
<tr><td>Position 2613-2620 of MCL1 3' UTR
hsa-miR-125b-5p</td><td>5' ...UUGUCUUACGCUUCUCUCAGGGA...
| | | | | | |
3' AGUGUUCAAUCCCAGAGUCCCU</td><td>8mer</td><td>-0.48</td><td>99</td><td>-0.04</td><td>5.312</td><td>0.95</td><td>Targetscan</td></tr>
<tr><td>Position 1532-1539 of STAT3 3' UTR
hsa-miR-125b-5p</td><td>5' ...ACGUGUCUGGUUGAGCUCAGGGA...
| | | | | | |
3' AGUGUUCAAUCCCAGAGUCCCU</td><td>8mer</td><td>-0.34</td><td>96</td><td>-0.30</td><td>4.716</td><td>0.93</td><td>Targetscan</td></tr>
<tr><td>Position 3380-3386 of EIF5A2 3' UTR
hsa-miR-125b-5p</td><td>5' ...UUUGAAUGCAUUGUUCUCAGGGC...
| | | | | | |
3' AGUGUUCAAUCCCAGAGUCCCU</td><td>7mer-m8</td><td>-0.32</td><td>96</td><td>-0.02</td><td>6.926</td><td>0.91</td><td>Targetscan</td></tr>
<tr><td>Position 142-148 of EIF4EBP1 3' UTR
hsa-miR-125b-5p</td><td>5' ...GCCCUUUCUCCCUCACUCAGGGC...
| | | | | | |
3' AGUGUUCAAUCCCAGAGUCCCU</td><td>7mer-m8</td><td>-0.34</td><td>96</td><td>-0.33</td><td>6.171</td><td>0.90</td><td>Targetscan</td></tr>
<tr><td>Position 2223-2229 of TP53 3' UTR
hsa-miR-125b-5p</td><td>5' …AAGACUUGUUUUAUGCUCAGGGU
| | | | | | |
3' AGUGUUCAAUCCCAGAGUCCCU</td><td>7mer-m8</td><td>N.A.</td><td>N.A.</td><td>N.A.</td><td>N.A.</td><td>N.A.</td><td>(14)</td></tr>
</table>

NA, not applicable; P_{CT}, probability of conserved targeting.

induced, at 48 h, a great increase in mean fluorescence intensity (MFI) due to MDC-positive staining, compared to untreated or miR-NC-transfected cells (Figures 4C and 4D; Figure S6). In detail, the fluorescence intensity was increased by 2.3-fold in miR-125b-transfected cells and by 1.6- to 1.7-fold in cells transfected with the other oligonucleotides. Notably, we also observed a modulation of autophagy markers; specifically, both Atg7 levels and LC3-II:I ratio were increased by miR-125b and chemically modified oligonucleotides if compared to the untreated cells, as clear indication of an augmented number of autophagosomes with the most significant upregulation found in cells treated with miR-125b-Omet (Figures 4E and 4F). Autophagic vesicle acidification was, instead, reverted at 72 h (Figure S7) when apoptosis occurrence was detected (Figures 4A and 4B).

Overall, these data suggest that autophagic flux, initially activated as a cytoprotective process able to inhibit apoptosis, is subsequently blocked with a consequent increase in apoptosis activation.

Senescence Occurrence after miR-125b Replacement

Based on the current knowledge that describes senescence as a process that can trigger autophagy as a mechanism of adaptation to the stress[25,32] and, at the same time, as a process that reduces cell reactivity to apoptotic stimuli,[33] we also investigated, at the same experimental conditions, the possible occurrence of senescence. The results obtained by cytochemical β-galactosidase assay indicated a marked activation of the senescence in correspondence with transfection with each of the three chemically modified oligonucleotides; in particular, already at 24 h (Figure 5A), about 60% senescence was observed after transfection with either miR-125b-Omet or miR-125b-LNA and 48% with the fluorinated miRNA. At the subsequent time (Figure 5B), also miR-125b induced an increase (58%) of senescent cells, and an additional potentiation (up to 70%) of the pro-senescence effect induced by miR-125b-Omet was found. As a confirmation of senescence induction, we also analyzed, by western blotting, the modulation of the main senescence mediators p21, p27, and p16 (Figure 5C). In detail, the extent of protein expression levels was significantly higher compared to control cells 24 h after miR-125b or miR-125b-Omet transfection, while miR-125b-LNA induced a protein increase to a lesser extent, limited to p21 and p27. Microscopic analysis of β-galactosidase-positive cells is shown in Figure 5D. β-galactosidase activity was almost completely abolished 72 h after transfection (Figure S8).

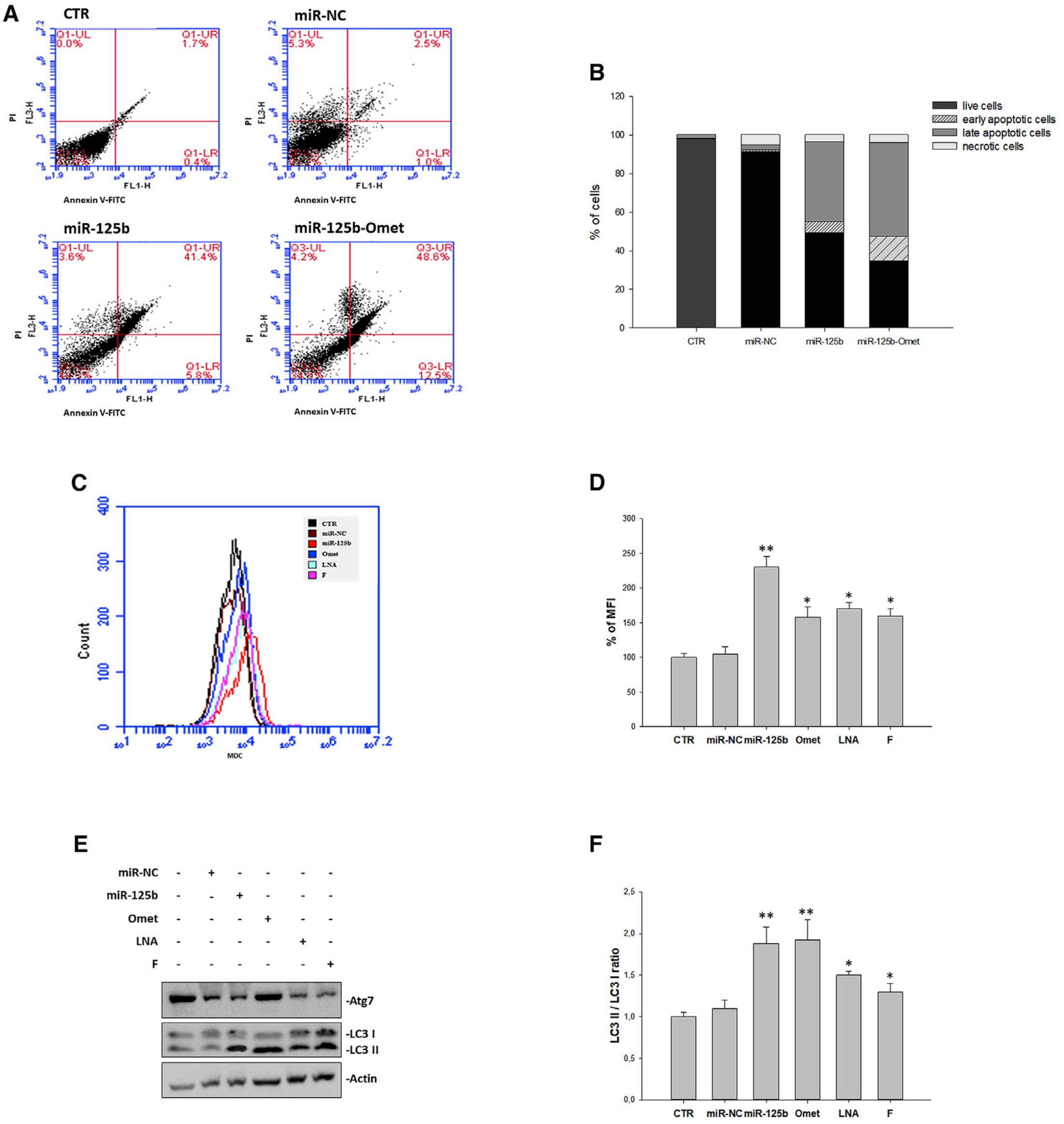

Figure 4. Analysis of Apoptosis and Autophagy Modulation

(A and B) The U266 cell line was transfected with miR-125b, miR-NC, or miR-125b-Omet at a final concentration of 100 nM for 72 h. After double staining with PI and Annexin V, as described in the Materials and Methods, cells were analyzed by flow cytometric analysis. (A) UL, upper left (necrosis); UR, high to right (in late apoptosis); LL, low left (viable); LR, lower right (early apoptosis); CTR, untreated cells. The data are representative of three different experiments that have always yielded similar results. The results are shown as cell percentage in the respective quadrants. (B) Stacked bar histogram represents the percentage of cells in the four quadrants. See also Figure S2 for 24- and 48-h fluorescence-activated cell sorting (FACS) analysis of apoptosis. (C) The U266 cell line was transfected with miR-125b, miR-NC, miR-125b-Omet (Omet), miR-125b-LNA (LNA), or miR-125b-2′F (F) at a final concentration of 100 nM for 48 h. After monodansylcadaverine (MDC) staining, cells were analyzed on the BD Accuri C6 flow cytometer. (D) The histogram shows the relative mean fluorescence intensity (MFI) with respect to the control sample. The results are shown as means ± SD for three independent experiments. $*p \leq 0.05$, $**p \leq 0.01$. See also Figure S7 for 72-h FACS analysis of autophagy. (E) The U266 cell line was transfected with miR-125b, miR-NC, miR-125b-Omet (Omet), miR-125b-LNA (LNA), or miR-125b-2′F (F) at a final concentration of 100 nM. After 48 h from transfection, the cells were collected for western blotting analysis. Subsequently, the expressions of Atg7 and LC3B I and II, was evaluated after blotting with specific antibodies, as described in the Materials and Methods. The actin protein was used as a loading control. (F) The histogram shows the ratio between the LC3 II and LC3 I forms. Densitometry analysis was performed using the ImageJ analysis tool. $*p \leq 0.05$, $**p \leq 0.01$.

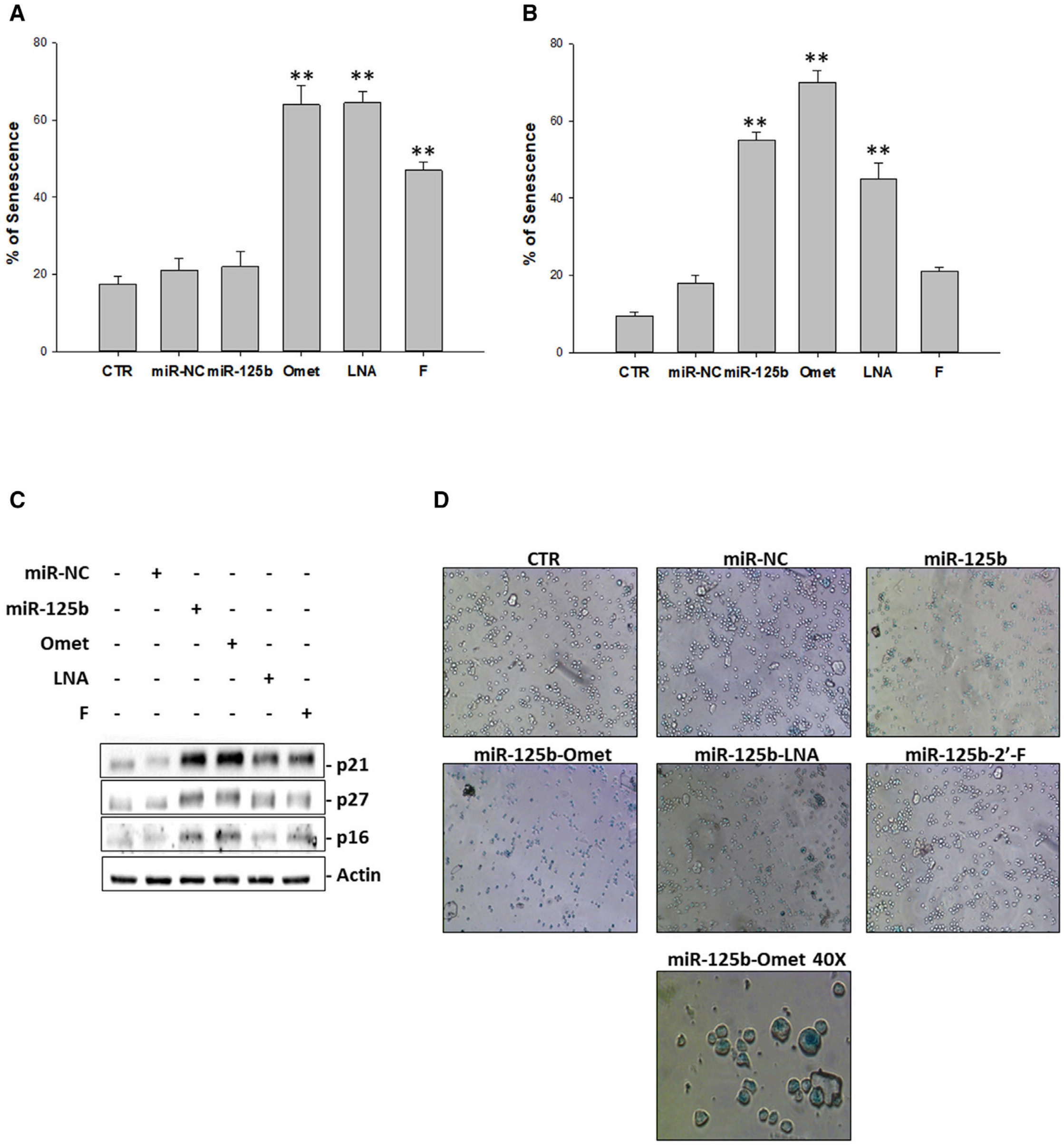

Figure 5. Analysis of Senescence

(A and B) Senescence-associated β-galactosidase assay. U266 cells were transfected with 100 nM miR-125b, miR-NC, miR-125b-Omet (Omet), miR-125b-LNA (LNA), or miR-125b-2′F (F). After 24 h (A) and 48 h (B) from transfection, cells were incubated with staining solution as described in the Materials and Methods and analyzed with a microplate reader to determine the amount of converted substrate at 570–595 nm. The histogram shows the percent of blue cells. The results are shown as means ± SD for three independent experiments. *$p \leq 0.05$, **$p \leq 0.01$. See also Figure S8 for 72-h analysis of β-galactosidase activity. (C) Western blotting analysis of senescence mediators. The U266 cell line was transfected with miR-125b, miR-NC, miR-125b-Omet (Omet), miR-125b-LNA (LNA), or miR-125b-2′F (F) at a final concentration of 100 nM. After 24 h from transfection, the cells were collected for western blotting analysis. Subsequently, the expressions of p21, p27, and p16 were evaluated after blotting with specific antibodies, as described in the Materials and Methods. The actin protein was used as a loading control. (D) Microscopic analysis of positive cells to β-galactosidase. The U266 cells were transfected with 100 nM miR-125b, miR-NC, miR-125b-Omet (Omet), miR-125b-LNA (LNA), or miR-125b-2′F (F). After 48 h from treatment, cells were observed with a bright-field microscope. Randomly selected fields were photographed at 10× and 40× magnifications.

Overall, early activation of senescence, in parallel with autophagy induction, could be recognized as the leading cause of apoptosis inhibition at 24 and 48 h. Indeed, the decreases in both processes at 72 h give way to apoptosis induction.

DISCUSSION

The miR-125 family is involved in a broad variety of cellular processes, i.e., differentiation, proliferation, and apoptosis, thereby playing a crucial role in different cancers, either as tumor suppressor or

promoter. Among the three homologs belonging to this family (hsa-miR-125a, hsa-miR-125b-1, and hsa-miR-125-2), miR-125b is transcribed by two loci placed on chromosomes 11q23 (hsa-miR-125b-1) and 21q21 (hsa-miR-125b-2).[58] Moreover, miR-125b-1 is implicated in a number of chromosomal translocations correlated with the occurrence of acute myeloid leukemia (AML) and B cell acute lymphoid leukemia (B-ALL) or myelodysplasia.[59,60] Despite that miR-125b dysregulation has been linked to tumor-promoting activity in different subtypes of leukemia[61–63] and to drug resistance in some cases of MM,[64] we have demonstrated that miR-125b acts as an oncosuppressor in MM by selective IRF4 inhibition.[24]

This study focused on the additional biochemical and functional characterization of MM cells after miR-125b-enforced expression. First, we selected the most suitable cell system, based on a model expressing low basal levels of miR-125b and exhibiting a significant antiproliferative response upon miRNA replacement. The U266 cell was the favored model presenting, together with SKMM-1 and OPM-2 cells, significantly lower miR-125b levels compared to RPMI 8226 and KMS-12 and, moreover, showing the most significant growth inhibition 24 and 48 h after miR-125b transfection. Most importantly, these findings allowed us to establish a correlation between basal miR-125b expression levels and the effectiveness of the miRNA-dependent growth-inhibitory effect by transient transfection. Indeed, RPMI 8226 cells viability was not significantly affected by transiently induced miR-125b ectopic expression, compared to previous results observed by stable miRNA transfection.[24] The antiproliferative effect of miR-125b has been reported in several studies involving solid tumors, such as breast,[65,66] ovarian,[67,68] cutaneous squamous cell,[69] hepatocellular[70–72] and bladder cancers,[73] osteosarcoma,[74] and melanoma.[75] By contrast, a clear tumorigenic effect was observed in endometrial carcinoma cell lines and in oligodendroglial,[76] pancreatic,[77] prostate,[78,79] and other cancers.

To better frame the molecular context, genetic profiling of MM cell lines was performed. The identification of genetic defects as common and rare variants in candidate regions of the human genome is essential to understand the etiology of complex human diseases. By mutational analysis, we identified deleterious mutations in several genes involved in cell proliferation and differentiation processes. In detail, U266 cells are mutated in MET, TP53, and BRAF genes; SKMM-1 cells are mutated in CSDE1 (upstream gene of NRAS), PTEN, and TP53; RPMI 8226 cells show a greater number of mutated genes, in particular ERBB4, PIK3CA, EGFR, KRAS, and TP53 (Table S1). Noteworthy, these three lines present SNVs in the TP53 gene, involving a complete or partial loss of transactivation activities, as we found in the TP53 variants database IARC TP53 Database (R18 version)[25] (Table S2). The antiproliferative role of p53 protein in response to various stressors and during physiological processes (e.g., senescence) makes it a primary target for inactivation in cancer.[80]

Based on the evident oncosuppressive role and in order to exploit this property in prospective studies *in vivo*, we designed a series of chemical modifications at the 2′-Ribose (2′-Omet, 2′-F, and LNA) to confer both nuclease resistance in the biological environment and increased stability and/or binding specificity of the miRNA-mRNA duplex. Indeed, RNA degradation mechanisms catalyzed by nucleases largely involve the 2′-OH of the ribose moiety,[81] so that its chemical modifications should result in an improved stability. Moreover, the insertion of a chemical moiety in the 2′ position favors the characteristic double-helix conformation of the miRNA-mRNA duplex. The antiproliferative effect induced by chemically modified oligonucleotides clearly demonstrated their efficacy in counteracting MM cell viability and, consequently, their role as miR-125b mimic with additional enhanced growth-inhibitory properties. Perturbations in the miRNA expression profile by miR-125b replacement were investigated in U266 cells, highlighting several up- and downregulated miRNAs, most of which are already characterized in cancer but not yet specifically in MM. Among these, we validated the ubiquitous oncosuppressive miR-34a, whose molecular mechanisms mediating anti-MM activity were already reported in our previous papers.[9,13,14,16,17]

Performing a comprehensive *in silico* search for miR-125b target prediction, we identified a panel of putative mRNA targets playing important roles in both the induction and progression of MM, as well as in maintaining the myeloma microenvironment. These targets were validated by western blot analysis. In detail, IL-6R, C-Raf, Akt, Bcl-2, Mcl-1, and EIF5A were clearly downregulated 48 h after miR-125b transfection, although their expression levels were significantly reduced or completely inhibited already at 24 h following miR-125b-Omet or miR-125b-LNA transfection. Silencing induced by Omet- and LNA-modified oligonucleotides was preserved or increased at the following time point. Of note, the oncogenic transcription factor STAT3 was significantly reduced already 24 h after miR-125b-Omet transfection, and it was further inhibited at the subsequent time; this effect was also established for cells transfected with miR-125b and, partly, also for miR-125b-LNA-transfected cells. Interestingly, the reduced expression of both the tumor suppressor p53 and the translation repressor protein 4EBP1, a key negative regulator of cell proliferation downstream of mTORC1, did not compromise the miRNA-mediated growth-inhibitory effect. Despite the significant p53 repression, the main reason for the global growth-inhibitory effect exerted by miR-125b may lie in the molecular context of the U266 cell line, where p53 mutation is associated with loss of function and its silencing does not produce the expected oncogenic effect, sometimes observed after miR-125b replacement.

It is well established that the BMM plays a prominent role in the biology of MM cells, since it elicits the production of cytokine, the enhancement of cell proliferation, and the resistance to chemotherapy by the activation of nuclear factor κB (NF-κB), PKC, PI3K/AKT, and JAK/STAT3 pathways through the largely characterized MM growth factor IL-6.[7] These pathways actively participate in transforming plasma cells. In detail, STAT3 has a great impact in the transcriptional activation of pro-survival mediators Bcl-2 and Mcl-1[82] that are, together with STAT3, miR-125b validated targets, as we have herein demonstrated. Therefore, STAT3 silencing further inhibits Bcl-2 and

Mcl-1 expression, already repressed by miR-125b itself (Figures 3C–3E). Moreover, being Bcl-2 an inducer of STAT3 activation,[83] miR-125b can exert its oncosuppressive role by interfering with this positive feedback loop. A recent study reported that IL-6-induced oncogenic effects are mediated by the direct repression of miR-34a by STAT3 and that the p53-dependent expression of miR-34a suppresses tumor progression by inhibiting the IL-6R/STAT3/miR-34a feedback loop, being IL-6R a direct target of miR-34a.[26] In this context, the up-regulation of miR-34a could be due, at least in part, to the simultaneous inhibition of IL-6R and STAT3 by miR-125b ectopic expression (Figure 3E).

In parallel with this oncosuppressor activity, we also observed an early miR-125b-induced cytoprotective effect, such as the accumulation and acidification of autophagosomes. Indeed, autophagy is often recognized as an anti-apoptotic process,[84] and this antagonistic effect is particularly apparent in U266 cells compared to other previously analyzed MM models, where a slight activation of apoptosis was detected at 48 h.[24] However, this escape from cell death was largely overcome by the induction of apoptosis after 72 h. Importantly, for the first time we have also established the induction of senescence, an additional cell death mechanism, upon miR-125b ectopic expression in MM cells. Accordingly, other authors have recently observed this effect following miR-125b replacement, though exclusively for melanoma cells.[85,86] It is well known that senescence can elicit autophagy as a mechanism of adaptation to stress,[25,32] reducing, at the same time, cell reactivity to apoptotic stimuli.[33] Overall, the observed cell death processes associated with miR-125b ectopic expression can be integrated as follows: early activation of senescence, in parallel with autophagy induction, likely lead to apoptosis inhibition at 24 and 48 h, while the progressive inactivation of both processes at 72 h could be the turning point toward apoptosis induction.

In conclusion, we have delineated the functional role of miR-125b in MM cell models, showing an oncosuppressive function based on the repression of multiple cancer-associated targets. For the first time, we identified a cross-regulation between miR-34a and miR-125b due to their inhibitory roles on the IL-6R/STAT3/miR-34a feedback loop, ultimately resulting in the stimulation of miR-34a expression. In this context, we demonstrated low miR-125b basal levels correlating with a higher efficient cell growth inhibition upon miRNA replacement. Moreover, we showed enhanced miR-125b-induced anticancer effects *in vitro* by the use of chemically modified analogs. miRNA profiling analysis allowed the identification of a pattern of differentially expressed miRNAs, thus opening future perspectives for the detection of new players in anti-MM activity. Finally, before apoptosis activation at 72 h and concurrently with autophagy onset, we identified early activation of senescence as the possible leading cause of apoptosis inhibition at 24 and 48 h from miR-125b ectopic expression. The anti-MM activity revealed *in vitro* by chemically modified miRNAs, inhibiting the expression of the main targets and prompting the functional effects of miR-125b, can represent a promising starting point for prospective preclinical investigations *in vivo* aimed at demonstrating the biological stability and therapeutic efficacy of these oligonucleotides.

MATERIALS AND METHODS

Cell Cultures

U266, RPMI 8226, KMS-12, OPM-2, and SKMM-1 MM cell lines, kindly provided by the Department of Clinical and Experimental Medicine of the University Magna Græcia, were grown in RPMI-1640 medium containing L-glutamine (Gibco, Life Technologies, Carlsbad, CA, USA), supplemented with heat-inactivated 20% fetal bovine serum (FBS) (Lonza Group, Switzerland), 20 mM HEPES, 100 U/mL penicillin, and 100 mg/mL streptomycin (Gibco, Life Technologies, Carlsbad, CA, USA) and incubated at 37°C in a 5% CO_2 atmosphere.

Next-Generation Sequencing-Based Expression Profiling in MM Cells

The genetic profiles of U266, RPMI 8226, and SKMM-1 cell lines were analyzed by sequencing using next-generation sequencing technologies. Genomic DNA was extracted using a Wizard Genomic DNA Purification Kit (Promega, USA), and quantification was performed with a fluorometer Qubit 3.0 (Life Technologies, Thermo Fisher Scientific, USA), according to the manufacturer's instructions. Amplicon re-sequencing strategy was used to create an amplicon library. An amount of 10 ng DNA was amplified using Ion AmpliSeq Library Kit 2.0 and an Ion AmpliSeq Community panel. The resulting amplicons were treated with FuPa Reagent to partially digest the primers. The amplicons were then ligated to Ion P1 Adapters with Ion Xpress Barcodes, using a Switch Solution and DNA Ligase, and purified using Agencourt AMPure XP Kit. The amplified library was quantified with Qubit 2.0 Fluorometer. Each DNA fragment is bound to a single ion sphere particle (ISP) with the P adaptor and multiplied by clonal amplification so each ISP is covered with many copies of the same DNA fragment. The clonal amplification by emulsion PCR was done using 100 pM diluted library on the Ion OneTouch 2 system with Ion PI Template OT2 200 Kit version (v.)2 chemistry (Life Technologies, Thermo Fisher Scientific, USA). Enrichment was conducted using the Ion OneTouch ES (Life Technologies, Thermo Fisher Scientific, USA). The prepared samples of Ion Sphere Particles were loaded onto an Ion 314 sequencing chip (Life Technologies) and sequenced on the Ion Personal Genoma Machine System using an Ion PGM Sequencing 200 Kit v.2 chemistry (200-bp read length, Life Technologies). All DNA samples were analyzed with an Ion AmpliSeq Panel for somatic mutation detection. This panel has primer pairs in a single pool for hotspots and targeted regions for 22 genes: KRAS, EGFR, BRAF, PIK3CA, AKT1, ERBB2, PTEN, NRAS, STK11, MAP2K1, ALK, DDR2, CTNNB1, MET, TP53, SMAD4, FBX7, FGFR3, NOTCH1, ERBB4, FGFR1, and FGFR2. The panel also includes primers for 3 additional amplicons covering target regions of the NRAS and ALK genes: NRAS exon 4 variants (p.117, p.146) and ALK variants (G1269A, p.S1206Y). Base calling and elaboration were executed by the Torrent Suite (Life Technologies, Thermo Fisher Scientific, USA) and Ion Reporter (https://ionreporter.thermofisher.com/ir/), which generated VCF files. To evaluate the reliability of the obtained result, the following parameters were considered: chip loading (percent of the chip wells physically filled with spheres,

ISP), enrichment (percent of spheres enriched with monoclonal libraries), and coverage (number of reads that include a given nucleotide in the reconstructed sequence). The data obtained are aligned against the human reference genome hg19 (filed by the Genome Reference Consortium).

2′-Omet, LNA, and 2′-F Oligonucleotide Synthesis and Purification

The 2′-Omet, LNA, and 2′-F oligonucleotides were synthesized by a Millipore Cyclone Plus DNA synthesizer at a 1-μmol scale, using commercially available 5′-O-(4,4'-dimethoxytrityl)-2′-O-methyl-3′-O-(2-cyanoethyl-N,N-diisopropyl) RNA phosphoramidite monomers and 2′-Omet RNA SynBaseCPG 1000/110 as solid-phase support (Link Technologies). The oligomers were detached from the support and deprotected by treatment with concentrated aqueous ammonia at 55°C for 12 h. The combined filtrates and washings were concentrated under reduced pressure, dissolved in H_2O, analyzed, and purified by anion-exchange high-performance liquid chromatography (HPLC) by a Nucleogel SAX column (Macherey-Nagel, 1000-8/46), using buffer A (20 mM KH_2PO_4/K_2HPO_4 aqueous solution [pH 7.0] containing 20% [v/v] CH_3CN) and buffer B (1 M KCl and 20 mM KH_2PO_4/K_2HPO_4 aqueous solution [pH 7.0] containing 20% [v/v] CH_3CN); a linear gradient from 0% to 100% B for 45 min and a flow rate of 1 mL/min were used. The purified oligomers were successively desalted by Sep-Pak C-18 cartridges (Waters) and characterized by ESI mass spectrometry.

In Vitro Transfection of MM Cell Lines

The miR-125b mimic was synthesized "in service" from the research laboratories of CEINGE (Biotecnologie Avanzate, Napoli, Italy). The sequence of the miR-125b mimic was 5′-UCCCUGAGACCCUAA CUUGUGA-3′. The sequence of the NC mimic was 5′-GCAA UUUGGCGUCCUCCACUAA-3′. The MM cell lines were seeded at a density of 112 × 10^3 cells/cm^2 in RPMI medium without antibiotics, and they were electroporated with miR-125b mimics and its modified analogs, at a final concentration of 100 nmol/L, using a Neon Transfection System (Invitrogen, Carlsbad, CA, USA; 1,050 V, for 30 ms, 1 pulse). An oligonucleotide with scrambled sequence (miRNA-NC) (Life Technologies, Carlsbad, CA, USA) at a concentration of 10 nmol/L was used as a control. Cell transfection efficiency was evaluated by flow cytometric analysis after fluorochrome-labeled oligonucleotide (FAM) (Invitrogen, Carlsbad, CA) transfection.

Cell Viability and Proliferation Assays

MM cell lines were electroporated, as previously described, and a viability assay was performed using Cellometer Auto 1000 (Nexcelom Bioscience, Lawrence, MA, USA) after trypan blue 0.2% staining (Lonza Walkersville), according to the manufacturer's instructions. Cell proliferation was evaluated by MTS colorimetric assay. CellTiter 96 AQueous One Solution Cell Proliferation assay (Promega, Milan, Italy) was used to determine cell growth, according to the manufacturer's instructions. The absorbance values at 492 nm were corrected by subtracting the average absorbance from the control wells containing only the cell medium. Each experiment was performed in triplicate and data were expressed as mean ± SD.

Western Blot Analysis

The U266 MM cell lines were electroporated with or without miR-125b mimics and its modified analogs, as previously described. For cell extract preparation, cells were washed twice with ice-cold PBS and BSA and centrifuged for 30 min at 4°C in 1 mL lysis buffer (1% Triton, 0.5% sodium deoxycholate, 0.1 M NaCl, 1 mM EDTA [pH 7.5], 10 mM Na_2HPO_4 [pH 7.4], 10 mM phenylmethylsulfonyl fluoride [PMSF], 25 mM benzamidin, 1 mM leupeptin, and 0.025 U/mL aprotinin). Lysates were spun at 13,000 × g for 10 min and supernatants were collected.[87] Protein concentration was determined by the Lowry method and compared with BSA standard curve. Equal amounts of cell proteins were separated by SDS-PAGE using TGX Stain-Free FastCast Acrylamide Solutions (Bio-Rad, CA, USA). The proteins were electro-transferred to nitrocellulose by Trans blot turbo (Bio-Rad, CA, USA) and reacted with the different monoclonal antibodies (mAbs). Stat3, p-Stat, and Mcl-2 mAbs were purchased from Santa Cruz Biotechnology (Dallas, TX, USA). 4-EBP1, p-4EBP1, Eif5A, p53, Craf, IL-6R, Akt, LC3B I and II, and Atg7 mAbs were purchased from Cell Signaling Technology (Beverly, MA, USA). Anti-actin mAb was purchased from Sigma-Aldrich (USA). After incubation with secondary antibodies, the signal was detected using Immobilon Western Chemiluminescent HRP Substrate (ECL, Millipore, MA, USA). Actin expression levels were used as the loading control in each sample. Densitometry analysis was performed using the ImageJ analysis tool.

Real-Time qPCR

Total RNA from MM cell lines was obtained by mirVana miRNA Isolation Kits (Life Technologies), according to the manufacturer's instructions. The quality and quantity of RNA were assessed by the NanoDrop ND-1000 Spectrophotometer (Thermo Scientific, Wilmington, DE, USA). Oligo-dT-primed cDNA was obtained using the High Capacity cDNA Reverse Transcription Kit (Applied Biosystems, CA, USA). The single-tube TaqMan miRNA assays (Ambion, Life Technologies, CA, USA) were used to detect and to quantify mature miR-125b and miR-34a, according to the manufacturer's instructions, by Real-time PCR Viia7 (Applied Biosystems, CA, USA). miR-125b and miR-34a expressions were normalized with RNU44 (Ambion, Life Technologies, CA, USA). Comparative real-time PCR was performed in triplicate, including no template controls, and relative expression was calculated using the comparative cross-threshold (Ct) method.

Flow Cytometric Analysis of Autophagy

The autophagy evaluation was performed after MDC (Sigma, USA) staining, a selective autophagolysosome marker.[88] The MM cell lines were electroporated in our experimental condition and incubated with 50 μM MDC for 10 min at 37°C and washed twice in PBS. The autophagic cell analysis was performed by flow cytometry (BD Accuri C6). For each sample, 1 × 10^4 events were acquired. Each

experiment was performed in triplicate and data were expressed as mean ± SD.

Senescence-Associated β-Galactosidase Assay

U266 cells, with miR-125b mimic- or modified analog-enforced expression, were fixed with 2% formaldehyde and 0.2% glutaraldehyde for 5 min at room temperature (RT), then incubated overnight with Staining Solution (5 mM $K_3Fe(CN)_6$, 5 mM $K_4Fe(CN)_6$, 150 mM NaCl, 2 mM $MgCl_2$, and X-Gal 1 mg/mL [Promega, USA]) in 0.2 M citric acid and 1 M Na phosphate buffer (pH 6.0). The cells were rinsed twice with PBS before microplate reader (iMark Microplate Absorbance Reader, Bio-Rad Laboratories, Hercules, CA, USA) analysis at 570–595 nm or microscopic examination. Concerning microscopic examination, the number of β-galactosidase active and senescent cells was determined by counting blue cells under a Nikon Eclipse TS100 model light microscope, and randomly selected fields were photographed at 10× and 40× magnifications.

Microarray Screening Assay

In miRNA screening assay, U266 cells were transfected with 100 nM miR-125b, miR-125b-omet, or miR-NC, and RNA was extracted 48 h later as described above. Starting from RNA 60 ng of each sample was synthesized cDNA with Megaplex RT Primers, Human Pool A v.2.1 (Applied Biosystems, CA, USA), according to the manufacturer's instructions. Human Pool A v.2.1 contains RT primers for 377 unique miRNAs and 4 controls. The miRNA expression profiling was subsequently performed using TaqMan Array Human MicroRNA A Cards v.2.0 (Applied Biosystems, CA, USA) and TaqMan Universal PCR Master Mix (Applied Biosystems, CA, USA), following the manufacturer's manuals. The assay was run on Viia 7 real-time PCR system (Applied Biosystems, CA, USA).

The Ct value of every miRNA was determined using Viia7 software (Applied Biosystems, CA, USA) and setting a threshold of 0.2. For calculation of the ΔCt for the miRNAs of interest, Ct values of every miRNA were normalized by RNU44 small nucleolar RNA (snoRNA) as an endogenous control. The relative miRNA expression was calculated with the ΔΔCt method. Fold change was calculated with the $2^{-\Delta\Delta Ct}$ method.

Statistical Analysis

Statistical analysis was performed using ANOVA. Significant differences were determined at $p \leq 0.05$ according to the Student's t test. Graphs were obtained using SigmaPlot v.11.0 (Systat Software, San Jose, CA, USA).

SUPPLEMENTAL INFORMATION

Supplemental Information can be found online at https://doi.org/10.1016/j.omtn.2019.02.023.

AUTHOR CONTRIBUTIONS

Conceived and Designed the Experiments, G.M., M.C., P. Tagliaferri, and P. Tassone; Performed the Experiments, M.R.Z., A. Lombardi, A. Grimaldi, C.F., M.R., A.M.C., H.K., M.T.D.M., A.F., and C.I.; Analyzed the Data, G.M., M.R.Z., A. Lombardi, A. Luce, and M.C.; Contributed Reagents/Materials/Analysis Tools, G.D.R., A. Galeone, A.V., M.D., and E.A.; Wrote the Paper, G.M., M.C., P. Tagliaferri, and P. Tassone.

ACKNOWLEDGMENTS

This work has been supported by the Italian Association for Cancer Research (AIRC) with "Special Program for Molecular Clinical Oncology–5 per mille," 2010/15 No. 9980, 2010/15 (PI: PT). The authors M.C. and E.A. would like to acknowledge networking support by the COST Action CA16122.

REFERENCES

1. Misso, G., Zappavigna, S., Castellano, M., De Rosa, G., Di Martino, M.T., Tagliaferri, P., Tassone, P., and Caraglia, M. (2013). Emerging pathways as individualized therapeutic target of multiple myeloma. Expert Opin. Biol. Ther. *13* (*Suppl 1*), S95–S109.
2. Roodman, G.D. (2009). Pathogenesis of myeloma bone disease. Leukemia *23*, 435–441.
3. Kyle, R.A., Durie, B.G., Rajkumar, S.V., Landgren, O., Blade, J., Merlini, G., Kröger, N., Einsele, H., Vesole, D.H., Dimopoulos, M., et al.; International Myeloma Working Group (2010). Monoclonal gammopathy of undetermined significance (MGUS) and smoldering (asymptomatic) multiple myeloma: IMWG consensus perspectives risk factors for progression and guidelines for monitoring and management. Leukemia *24*, 1121–1127.
4. Kyle, R.A., Remstein, E.D., Therneau, T.M., Dispenzieri, A., Kurtin, P.J., Hodnefield, J.M., Larson, D.R., Plevak, M.F., Jelinek, D.F., Fonseca, R., et al. (2007). Clinical course and prognosis of smoldering (asymptomatic) multiple myeloma. N. Engl. J. Med. *356*, 2582–2590.
5. Morgan, G.J., Walker, B.A., and Davies, F.E. (2012). The genetic architecture of multiple myeloma. Nat. Rev. Cancer *12*, 335–348.
6. Podar, K., Tai, Y.T., Lin, B.K., Narsimhan, R.P., Sattler, M., Kijima, T., Salgia, R., Gupta, D., Chauhan, D., and Anderson, K.C. (2002). Vascular endothelial growth factor-induced migration of multiple myeloma cells is associated with beta 1 integrin- and phosphatidylinositol 3-kinase-dependent PKC alpha activation. J. Biol. Chem. *277*, 7875–7881.
7. Hideshima, T., Mitsiades, C., Tonon, G., Richardson, P.G., and Anderson, K.C. (2007). Understanding multiple myeloma pathogenesis in the bone marrow to identify new therapeutic targets. Nat. Rev. Cancer *7*, 585–598.
8. Rossi, M., Di Martino, M.T., Morelli, E., Leotta, M., Rizzo, A., Grimaldi, A., Misso, G., Tassone, P., and Caraglia, M. (2012). Molecular targets for the treatment of multiple myeloma. Curr. Cancer Drug Targets *12*, 757–767.
9. Misso, G., Di Martino, M.T., De Rosa, G., Farooqi, A.A., Lombardi, A., Campani, V., Zarone, M.R., Gullà, A., Tagliaferri, P., Tassone, P., and Caraglia, M. (2014). Mir-34: a new weapon against cancer? Mol. Ther. Nucleic Acids *3*, e194.
10. Misso, G., Zarone, M.R., Grimaldi, A., Di Martino, M.T., Lombardi, A., Kawasaki, H., Stiuso, P., Tassone, P., Tagliaferri, P., and Caraglia, M. (2017). Non coding RNAs: a new avenue for the self-tailoring of blood cancer treatment. Curr. Drug Targets *18*, 35–55.
11. Grimaldi, A., Zarone, M.R., Irace, C., Zappavigna, S., Lombardi, A., Kawasaki, H., Caraglia, M., and Misso, G. (2018). Non-coding RNAs as a new dawn in tumor diagnosis. Semin. Cell Dev. Biol. *78*, 37–50.
12. Garzon, R., Marcucci, G., and Croce, C.M. (2010). Targeting microRNAs in cancer: rationale, strategies and challenges. Nat. Rev. Drug Discov. *9*, 775–789.
13. Zarone, M.R., Misso, G., Grimaldi, A., Zappavigna, S., Russo, M., Amler, E., Di Martino, M.T., Amodio, N., Tagliaferri, P., Tassone, P., and Caraglia, M. (2017). Evidence of novel miR-34a-based therapeutic approaches for multiple myeloma treatment. Sci. Rep. *7*, 17949.
14. Di Martino, M.T., Campani, V., Misso, G., Gallo Cantafio, M.E., Gullà, A., Foresta, U., Guzzi, P.H., Castellano, M., Grimaldi, A., Gigantino, V., et al. (2014). In vivo activity

of miR-34a mimics delivered by stable nucleic acid lipid particles (SNALPs) against multiple myeloma. PLoS ONE *9*, e90005.

15. Amodio, N., Di Martino, M.T., Foresta, U., Leone, E., Lionetti, M., Leotta, M., Gullà, A.M., Pitari, M.R., Conforti, F., Rossi, M., et al. (2012). miR-29b sensitizes multiple myeloma cells to bortezomib-induced apoptosis through the activation of a feedback loop with the transcription factor Sp1. Cell Death Dis. *3*, e436.

16. Scognamiglio, I., Di Martino, M.T., Campani, V., Virgilio, A., Galeone, A., Gullà, A., Gallo Cantafio, M.E., Misso, G., Tagliaferri, P., Tassone, P., et al. (2014). Transferrin-conjugated SNALPs encapsulating 2′-O-methylated miR-34a for the treatment of multiple myeloma. BioMed Res. Int. *2014*, 217365.

17. Di Martino, M.T., Leone, E., Amodio, N., Foresta, U., Lionetti, M., Pitari, M.R., Cantafio, M.E., Gullà, A., Conforti, F., Morelli, E., et al. (2012). Synthetic miR-34a mimics as a novel therapeutic agent for multiple myeloma: in vitro and in vivo evidence. Clin. Cancer Res. *18*, 6260–6270.

18. Di Martino, M.T., Gullà, A., Cantafio, M.E., Lionetti, M., Leone, E., Amodio, N., Guzzi, P.H., Foresta, U., Conforti, F., Cannataro, M., et al. (2013). In vitro and in vivo anti-tumor activity of miR-221/222 inhibitors in multiple myeloma. Oncotarget *4*, 242–255.

19. Leone, E., Morelli, E., Di Martino, M.T., Amodio, N., Foresta, U., Gullà, A., Rossi, M., Neri, A., Giordano, A., Munshi, N.C., et al. (2013). Targeting miR-21 inhibits in vitro and in vivo multiple myeloma cell growth. Clin. Cancer Res. *19*, 2096–2106.

20. Morelli, E., Biamonte, L., Federico, C., Amodio, N., Di Martino, M.T., Gallo Cantafio, M.E., Manzoni, M., Scionti, F., Samur, M.K., Gullà, A., et al. (2018). Therapeutic vulnerability of multiple myeloma to MIR17PTi, a first-in-class inhibitor of pri-miR-17-92. Blood *132*, 1050–1063.

21. Le, M.T.N., Teh, C., Shyh-Chang, N., Xie, H., Zhou, B., Korzh, V., Lodish, H.F., and Lim, B. (2009). MicroRNA-125b is a novel negative regulator of p53. Genes Dev. *23*, 862–876.

22. Kumar, M., Lu, Z., Takwi, A.A., Chen, W., Callander, N.S., Ramos, K.S., Young, K.H., and Li, Y. (2011). Negative regulation of the tumor suppressor p53 gene by microRNAs. Oncogene *30*, 843–853.

23. Gururajan, M., Haga, C.L., Das, S., Leu, C.M., Hodson, D., Josson, S., Turner, M., and Cooper, M.D. (2010). MicroRNA 125b inhibition of B cell differentiation in germinal centers. Int. Immunol. *22*, 583–592.

24. Morelli, E., Leone, E., Cantafio, M.E., Di Martino, M.T., Amodio, N., Biamonte, L., Gullà, A., Foresta, U., Pitari, M.R., Botta, C., et al. (2015). Selective targeting of IRF4 by synthetic microRNA-125b-5p mimics induces anti-multiple myeloma activity in vitro and in vivo. Leukemia *29*, 2173–2183.

25. Gerland, L.M., Peyrol, S., Lallemand, C., Branche, R., Magaud, J.P., and Ffrench, M. (2003). Association of increased autophagic inclusions labeled for beta-galactosidase with fibroblastic aging. Exp. Gerontol. *38*, 887–895.

26. Stroikin, Y., Dalen, H., Brunk, U.T., and Terman, A. (2005). Testing the “garbage” accumulation theory of ageing: mitotic activity protects cells from death induced by inhibition of autophagy. Biogerontology *6*, 39–47.

27. Maiuri, M.C., Zalckvar, E., Kimchi, A., and Kroemer, G. (2007). Self-eating and self-killing: crosstalk between autophagy and apoptosis. Nat. Rev. Mol. Cell Biol. *8*, 741–752.

28. Seluanov, A., Gorbunova, V., Falcovitz, A., Sigal, A., Milyavsky, M., Zurer, I., Shohat, G., Goldfinger, N., and Rotter, V. (2001). Change of the death pathway in senescent human fibroblasts in response to DNA damage is caused by an inability to stabilize p53. Mol. Cell. Biol. *21*, 1552–1564.

29. Yeo, E.J., Hwang, Y.C., Kang, C.M., Choy, H.E., and Park, S.C. (2000). Reduction of UV-induced cell death in the human senescent fibroblasts. Mol. Cells *10*, 415–422.

30. Kole, R., Krainer, A.R., and Altman, S. (2012). RNA therapeutics: beyond RNA interference and antisense oligonucleotides. Nat. Rev. Drug Discov. *11*, 125–140.

31. Khvorova, A., and Watts, J.K. (2017). The chemical evolution of oligonucleotide therapies of clinical utility. Nat. Biotechnol. *35*, 238–248.

32. Vicencio, J.M., Galluzzi, L., Tajeddine, N., Ortiz, C., Criollo, A., Tasdemir, E., Morselli, E., Ben Younes, A., Maiuri, M.C., Lavandero, S., and Kroemer, G. (2008). Senescence, apoptosis or autophagy? When a damaged cell must decide its path–a mini-review. Gerontology *54*, 92–99.

33. Rochette, P.J., and Brash, D.E. (2008). Progressive apoptosis resistance prior to senescence and control by the anti-apoptotic protein BCL-xL. Mech. Ageing Dev. *129*, 207–214.

34. Bouaoun, L., Sonkin, D., Ardin, M., Hollstein, M., Byrnes, G., Zavadil, J., and Olivier, M. (2016). TP53 Variations in Human Cancers: New Lessons from the IARC TP53 Database and Genomics Data. Hum. Mutat. *37*, 865–876.

35. Li, X.X., Gao, S.Y., Wang, P.Y., Zhou, X., Li, Y.J., Yu, Y., Yan, Y.F., Zhang, H.H., Lv, C.J., Zhou, H.H., and Xie, S.Y. (2015). Reduced expression levels of let-7c in human breast cancer patients. Oncol. Lett. *9*, 1207–1212.

36. Nadiminty, N., Tummala, R., Lou, W., Zhu, Y., Shi, X.B., Zou, J.X., Chen, H., Zhang, J., Chen, X., Luo, J., et al. (2012). MicroRNA let-7c is downregulated in prostate cancer and suppresses prostate cancer growth. PLoS ONE *7*, e32832.

37. Xie, Y., Zhang, H., Guo, X.J., Feng, Y.C., He, R.Z., Li, X., Yu, S., Zhao, Y., Shen, M., Zhu, F., et al. (2018). Let-7c inhibits cholangiocarcinoma growth but promotes tumor cell invasion and growth at extrahepatic sites. Cell Death Dis. *9*, 249.

38. Hou, L., Luo, P., Ma, Y., Jia, C., Yu, F., Lv, Z., Wu, C., and Fu, D. (2017). MicroRNA-125a-3p downregulation correlates with tumorigenesis and poor prognosis in patients with non-small cell lung cancer. Oncol. Lett. *14*, 4441–4448.

39. Xu, X., Lv, Y.G., Yan, C.Y., Yi, J., and Ling, R. (2016). Enforced expression of hsa-miR-125a-3p in breast cancer cells potentiates docetaxel sensitivity via modulation of BRCA1 signaling. Biochem. Biophys. Res. Commun. *479*, 893–900.

40. Jin, C., Li, M., Ouyang, Y., Tan, Z., and Jiang, Y. (2017). MiR-424 functions as a tumor suppressor in glioma cells and is down-regulated by DNA methylation. J. Neurooncol. *133*, 247–255.

41. Liu, J., Gu, Z., Tang, Y., Hao, J., Zhang, C., and Yang, X. (2018). Tumour-suppressive microRNA-424-5p directly targets CCNE1 as potential prognostic markers in epithelial ovarian cancer. Cell Cycle *17*, 309–318.

42. Wei, S., Li, Q., Li, Z., Wang, L., Zhang, L., and Xu, Z. (2016). miR-424-5p promotes proliferation of gastric cancer by targeting Smad3 through TGF-β signaling pathway. Oncotarget *7*, 75185–75196.

43. Yao, H., Liu, X., Chen, S., Xia, W., and Chen, X. (2015). Decreased expression of serum miR-424 correlates with poor prognosis of patients with hepatocellular carcinoma. Int. J. Clin. Exp. Pathol. *8*, 14830–14835.

44. Lu, Y.F., Zhang, L., Waye, M.M., Fu, W.M., and Zhang, J.F. (2015). MiR-218 mediates tumorigenesis and metastasis: Perspectives and implications. Exp. Cell Res. *334*, 173–182.

45. Li, Q., Ding, C., Chen, C., Zhang, Z., Xiao, H., Xie, F., Lei, L., Chen, Y., Mao, B., Jiang, M., et al. (2014). miR-224 promotion of cell migration and invasion by targeting Homeobox D 10 gene in human hepatocellular carcinoma. J. Gastroenterol. Hepatol. *29*, 835–842.

46. Cui, R., Kim, T., Fassan, M., Meng, W., Sun, H.L., Jeon, Y.J., Vicentini, C., Tili, E., Peng, Y., Scarpa, A., et al. (2015). MicroRNA-224 is implicated in lung cancer pathogenesis through targeting caspase-3 and caspase-7. Oncotarget *6*, 21802–21815.

47. Wang, M., Deng, X., Ying, Q., Jin, T., Li, M., and Liang, C. (2015). MicroRNA-224 targets ERG2 and contributes to malignant progressions of meningioma. Biochem. Biophys. Res. Commun. *460*, 354–361.

48. Ma, D., Tao, X., Gao, F., Fan, C., and Wu, D. (2012). miR-224 functions as an onco-miRNA in hepatocellular carcinoma cells by activating AKT signaling. Oncol. Lett. *4*, 483–488.

49. Li, Z., Zhang, G., Li, D., Jie, Z., Chen, H., Xiong, J., Liu, Y., Cao, Y., Jiang, M., Le, Z., and Tan, S. (2015). Methylation-associated silencing of miR-495 inhibit the migration and invasion of human gastric cancer cells by directly targeting PRL-3. Biochem. Biophys. Res. Commun. *456*, 344–350.

50. Tan, M., Mu, X., Liu, Z., Tao, L., Wang, J., Ge, J., and Qiu, J. (2017). microRNA-495 promotes bladder cancer cell growth and invasion by targeting phosphatase and tensin homolog. Biochem. Biophys. Res. Commun. *483*, 867–873.

51. Bai, Z., Wang, J., Wang, T., Li, Y., Zhao, X., Wu, G., Yang, Y., Deng, W., and Zhang, Z. (2017). The MiR-495/Annexin A3/P53 Axis Inhibits the Invasion and EMT of Colorectal Cancer Cells. Cell. Physiol. Biochem. *44*, 1882–1895.

52. Zhang, H., Li, S., Yang, X., Qiao, B., Zhang, Z., and Xu, Y. (2016). miR-539 inhibits prostate cancer progression by directly targeting SPAG5. J. Exp. Clin. Cancer Res. *35*, 60.

53. Yang, Z.X., Zhang, B., Wei, J., Jiang, G.Q., Wu, Y.L., Leng, B.J., and Xing, C.G. (2018). MiR-539 inhibits proliferation and migration of triple-negative breast cancer cells by down-regulating LAMA4 expression. Cancer Cell Int. *18*, 16.

54. Wu, Y., You, J., Li, F., Wang, F., and Wang, Y. (2018). MicroRNA-542-3p suppresses tumor cell proliferation via targeting Smad2 inhuman osteosarcoma. Oncol. Lett. *15*, 6895–6902.

55. Yang, C., Wang, M.H., Zhou, J.D., and Chi, Q. (2017). Upregulation of miR-542-3p inhibits the growth and invasion of human colon cancer cells through PI3K/AKT/survivin signaling. Oncol. Rep. *38*, 3545–3553.

56. Dong, P., Ihira, K., Xiong, Y., Watari, H., Hanley, S.J., Yamada, T., Hosaka, M., Kudo, M., Yue, J., and Sakuragi, N. (2016). Reactivation of epigenetically silenced miR-124 reverses the epithelial-to-mesenchymal transition and inhibits invasion in endometrial cancer cells via the direct repression of IQGAP1 expression. Oncotarget *7*, 20260–20270.

57. Rokavec, M., Öner, M.G., Li, H., Jackstadt, R., Jiang, L., Lodygin, D., Kaller, M., Horst, D., Ziegler, P.K., Schwitalla, S., et al. (2014). IL-6R/STAT3/miR-34a feedback loop promotes EMT-mediated colorectal cancer invasion and metastasis. J. Clin. Invest. *124*, 1853–1867.

58. Rodriguez, A., Griffiths-Jones, S., Ashurst, J.L., and Bradley, A. (2004). Identification of mammalian microRNA host genes and transcription units. Genome Res. *14* (10A), 1902–1910.

59. Bousquet, M., Quelen, C., Rosati, R., Mansat-De Mas, V., La Starza, R., Bastard, C., Lippert, E., Talmant, P., Lafage-Pochitaloff, M., Leroux, D., et al. (2008). Myeloid cell differentiation arrest by miR-125b-1 in myelodysplastic syndrome and acute myeloid leukemia with the t(2;11)(p21;q23) translocation. J. Exp. Med. *205*, 2499–2506.

60. Chapiro, E., Russell, L.J., Struski, S., Cavé, H., Radford-Weiss, I., Valle, V.D., Lachenaud, J., Brousset, P., Bernard, O.A., Harrison, C.J., and Nguyen-Khac, F. (2010). A new recurrent translocation t(11;14)(q24;q32) involving IGH@ and miR-125b-1 in B-cell progenitor acute lymphoblastic leukemia. Leukemia *24*, 1362–1364.

61. Liu, J., Guo, B., Chen, Z., Wang, N., Iacovino, M., Cheng, J., Roden, C., Pan, W., Khan, S., Chen, S., et al. (2017). *miR-125b* promotes *MLL-AF9*-driven murine acute myeloid leukemia involving a *VEGFA*-mediated non-cell-intrinsic mechanism. Blood *129*, 1491–1502.

62. Li, Q., Wu, Y., Zhang, Y., Sun, H., Lu, Z., Du, K., Fang, S., and Li, W. (2016). miR-125b regulates cell progression in chronic myeloid leukemia via targeting BAK1. Am. J. Transl. Res. *8*, 447–459.

63. Bousquet, M., Harris, M.H., Zhou, B., and Lodish, H.F. (2010). MicroRNA miR-125b causes leukemia. Proc. Natl. Acad. Sci. USA *107*, 21558–21563.

64. Murray, M.Y., Rushworth, S.A., Zaitseva, L., Bowles, K.M., and Macewan, D.J. (2013). Attenuation of dexamethasone-induced cell death in multiple myeloma is mediated by miR-125b expression. Cell Cycle *12*, 2144–2153.

65. Li, W., Duan, R., Kooy, F., Sherman, S.L., Zhou, W., and Jin, P. (2009). Germline mutation of microRNA-125a is associated with breast cancer. J. Med. Genet. *46*, 358–360.

66. Scott, G.K., Goga, A., Bhaumik, D., Berger, C.E., Sullivan, C.S., and Benz, C.C. (2007). Coordinate suppression of ERBB2 and ERBB3 by enforced expression of micro-RNA miR-125a or miR-125b. J. Biol. Chem. *282*, 1479–1486.

67. Cowden Dahl, K.D., Dahl, R., Kruichak, J.N., and Hudson, L.G. (2009). The epidermal growth factor receptor responsive miR-125a represses mesenchymal morphology in ovarian cancer cells. Neoplasia *11*, 1208–1215.

68. Guan, Y., Yao, H., Zheng, Z., Qiu, G., and Sun, K. (2011). MiR-125b targets BCL3 and suppresses ovarian cancer proliferation. Int. J. Cancer *128*, 2274–2283.

69. Xu, N., Zhang, L., Meisgen, F., Harada, M., Heilborn, J., Homey, B., Grandér, D., Ståhle, M., Sonkoly, E., and Pivarcsi, A. (2012). MicroRNA-125b down-regulates matrix metallopeptidase 13 and inhibits cutaneous squamous cell carcinoma cell proliferation, migration, and invasion. J. Biol. Chem. *287*, 29899–29908.

70. Bi, Q., Tang, S., Xia, L., Du, R., Fan, R., Gao, L., Jin, J., Liang, S., Chen, Z., Xu, G., et al. (2012). Ectopic expression of MiR-125a inhibits the proliferation and metastasis of hepatocellular carcinoma by targeting MMP11 and VEGF. PLoS ONE *7*, e40169.

71. Liang, L., Wong, C.M., Ying, Q., Fan, D.N., Huang, S., Ding, J., Yao, J., Yan, M., Li, J., Yao, M., et al. (2010). MicroRNA-125b suppressesed human liver cancer cell proliferation and metastasis by directly targeting oncogene LIN28B2. Hepatology *52*, 1731–1740.

72. Jia, H.Y., Wang, Y.X., Yan, W.T., Li, H.Y., Tian, Y.Z., Wang, S.M., and Zhao, H.L. (2012). MicroRNA-125b functions as a tumor suppressor in hepatocellular carcinoma cells. Int. J. Mol. Sci. *13*, 8762–8774.

73. Huang, L., Luo, J., Cai, Q., Pan, Q., Zeng, H., Guo, Z., Dong, W., Huang, J., and Lin, T. (2011). MicroRNA-125b suppresses the development of bladder cancer by targeting E2F3. Int. J. Cancer *128*, 1758–1769.

74. Liu, L.H., Li, H., Li, J.P., Zhong, H., Zhang, H.C., Chen, J., and Xiao, T. (2011). miR-125b suppresses the proliferation and migration of osteosarcoma cells through down-regulation of STAT3. Biochem. Biophys. Res. Commun. *416*, 31–38.

75. Kappelmann, M., Kuphal, S., Meister, G., Vardimon, L., and Bosserhoff, A.K. (2013). MicroRNA miR-125b controls melanoma progression by direct regulation of c-Jun protein expression. Oncogene *32*, 2984–2991.

76. Nelson, P.T., Baldwin, D.A., Kloosterman, W.P., Kauppinen, S., Plasterk, R.H., and Mourelatos, Z. (2006). RAKE and LNA-ISH reveal microRNA expression and localization in archival human brain. RNA *12*, 187–191.

77. Bloomston, M., Frankel, W.L., Petrocca, F., Volinia, S., Alder, H., Hagan, J.P., Liu, C.G., Bhatt, D., Taccioli, C., and Croce, C.M. (2007). MicroRNA expression patterns to differentiate pancreatic adenocarcinoma from normal pancreas and chronic pancreatitis. JAMA *297*, 1901–1908.

78. Shi, X.B., Xue, L., Yang, J., Ma, A.H., Zhao, J., Xu, M., Tepper, C.G., Evans, C.P., Kung, H.J., and deVere White, R.W. (2007). An androgen-regulated miRNA suppresses Bak1 expression and induces androgen-independent growth of prostate cancer cells. Proc. Natl. Acad. Sci. USA *104*, 19983–19988.

79. Shi, X.B., Xue, L., Ma, A.H., Tepper, C.G., Kung, H.J., and White, R.W. (2011). miR-125b promotes growth of prostate cancer xenograft tumor through targeting pro-apoptotic genes. Prostate *71*, 538–549.

80. Levine, A.J. (1997). p53, the cellular gatekeeper for growth and division. Cell *88*, 323–331.

81. Nicholson, A.W. (2014). Ribonuclease III mechanisms of double-stranded RNA cleavage. Wiley Interdiscip. Rev. RNA *5*, 31–48.

82. Bhattacharya, S., Ray, R.M., and Johnson, L.R. (2005). STAT3-mediated transcription of Bcl-2, Mcl-1 and c-IAP2 prevents apoptosis in polyamine-depleted cells. Biochem. J. *392*, 335–344.

83. Kang, J., Chong, S.J., Ooi, V.Z., Vali, S., Kumar, A., Kapoor, S., Abbasi, T., Hirpara, J.L., Loh, T., Goh, B.C., and Pervaiz, S. (2015). Overexpression of Bcl-2 induces STAT-3 activation via an increase in mitochondrial superoxide. Oncotarget *6*, 34191–34205.

84. Grimaldi, A., Santini, D., Zappavigna, S., Lombardi, A., Misso, G., Boccellino, M., Desiderio, V., Vitiello, P.P., Di Lorenzo, G., Zoccoli, A., et al. (2015). Antagonistic effects of chloroquine on autophagy occurrence potentiate the anticancer effects of everolimus on renal cancer cells. Cancer Biol. Ther. *16*, 567–579.

85. Nyholm, A.M., Lerche, C.M., Manfé, V., Biskup, E., Johansen, P., Morling, N., Thomsen, B.M., Glud, M., and Gniadecki, R. (2014). miR-125b induces cellular senescence in malignant melanoma. BMC Dermatol. *14*, 8.

86. Glud, M., Manfé, V., Biskup, E., Holst, L., Dirksen, A.M., Hastrup, N., Nielsen, F.C., Drzewiecki, K.T., and Gniadecki, R. (2011). MicroRNA miR-125b induces senescence in human melanoma cells. Melanoma Res. *21*, 253–256.

87. Sirangelo, I., Iannuzzi, C., Vilasi, S., Irace, G., Giuberti, G., Misso, G., D'Alessandro, A., Abbruzzese, A., and Caraglia, M. (2009). W7FW14F apomyoglobin amyloid aggregates-mediated apoptosis is due to oxidative stress and AKT inactivation caused by Ras and Rac. J. Cell. Physiol. *221*, 412–423.

88. Zappavigna, S., Lombardi, A., Misso, G., Grimaldi, A., and Caraglia, M. (2017). Measurement of Autophagy by Flow Cytometry. Methods Mol. Biol. *1553*, 209–216.

89. Miranda, K.C., Huynh, T., Tay, Y., Ang, Y.S., Tam, W.L., Thomson, A.M., Lim, B., and Rigoutsos, I. (2006). A pattern-based method for the identification of MicroRNA binding sites and their corresponding heteroduplexes. Cell *126*, 1203–1217.

OMTN, Volume *16*

Supplemental Information

miR-125b Upregulates miR-34a and Sequentially Activates Stress Adaption and Cell Death Mechanisms in Multiple Myeloma

Gabriella Misso, Mayra Rachele Zarone, Angela Lombardi, Anna Grimaldi, Alessia Maria Cossu, Carmela Ferri, Margherita Russo, Daniela Cristina Vuoso, Amalia Luce, Hiromichi Kawasaki, Maria Teresa Di Martino, Antonella Virgilio, Agostino Festa, Aldo Galeone, Giuseppe De Rosa, Carlo Irace, Massimo Donadelli, Alois Necas, Evzen Amler, Pierosandro Tagliaferri, Pierfrancesco Tassone, and Michele Caraglia

Table S1. Mutational analysis of MM cells using Ion Reporter Browser.

A

Genes	Locus	Genotype	Type	Location	Amino acid change	Coding	Exon	p-value	Variant effect	Allele Source
MET	chr7:116411990	C/T	SNV	exonic	p.Thr1010Ile	c.3029C>T	14	0.00001	missense	Hotspot
BRAF	chr7:140453132	T/A	SNV	exonic	p.Lys601Asn	c.1803A>T	15	0.00001	missense	Hotspot
TP53	chr17:7578448	G/G	INDEL	exonic	p.Ala161fs	c.481delG	5	0.00001	frameshift deletion	Novel
TP53	chr17:7578449	T/T	SNV	exonic	p.Ala161Thr	c.481G>A	5	0.00001	missense	Hotspot

B

Genes	Locus	Genotype	Type	Location	Amino acid change	Coding	Exon	p-value	Variant effect	Allele Source
CSDE1	chr1:115258747	CC/TC	SNV	Exonic, downstream	p.Gly12Asp	c.35G>A	2	0.00001	missense	Hotspot
PTEN	chr10:89685268	A/C	SNV	splicesite_5			3	0.00001		Hotspot
TP53	chr17:7578368	CACCATCGCTATCTGAGCAGCGCTCATGGTGGGGGCAGCGCCTCACA/CACCATCGCTATCTGAGCAGCGCTCATGGTGGGGGCAGCCCTCACA	INDEL	splicesite_3			5	0.00001		Novel
TP53	chr17:7578407	GC/CC	SNV	exonic	p.Arg175Gly	c.523C>G	5	0.00001	missense	Hotspot
TP53	chr17:7579470	CGG/CGC	SNV	exonic	p.Pro72Arg	c.215C>G	4	0.00001	missense	Novel

C

Genes	Locus	Genotype	Type	Location	Amino acid change	Coding	Exon	p-value	Variant effect	Allele Source
ERBB4	chr2:212589802	A/C	SNV	exonic	p.Phe247Cys	c.740T>G	6	0.00001	missense	Novel
PIK3CA	chr3:178936091	G/A	SNV	exonic	p.Glu545Lys	c.1633G>A	10	0.00001	missense	Hotspot
EGFR	chr7:55242482	CATCTCCGAAAGCCAACAAGGAAAT/TATCTCCGAAAGCCAACAAGGAAAT	SNV	exonic	p.Thr751Ile	c.2252C>T	19	0.00001	missense	Hotspot
KRAS	chr12:25398284		SNV	exonic	p.Gly12Ala	c.35G>C	2	0.00001	missense	Hotspot
TP53	chr17:7577085		SNV	exonic	p.Glu285Lys	c.853G>A	8	0.00001	missense	Hotspot

A: U266 cell line; B: SKMM-1 cell line; C: RPMI 8226 cell line; SNV: single nucleotide variants; INDEL: deletion; Variant effect : effect of the variant on the coding sequence; Hotspot: variant overlaps a hotspot file; Novel: not known variant.

Table S2. TP53 variants analysis.

	cDNA description	Protein description	Exon number	Effect	TA Class
**U266**	c.481G>A	p.A161T	5-exon	missense	Partially functional
**U266**	c.481delG	p.A161fs	5-exon	Frameshift	NA
**SKMM-1**	c.215C>G	p.P72R	4-exon	missense	NA
**SKMM-1**	c.523C>G	p.R175G	5-exon	missense	Non-functional
**RPMI 8226**	c.853G>A	p.E285K	8-exon	missense	Non-functional

TA Class: transactivation activities; NA: Not Available

Supplementary Figure legends

Figure S1. Evaluation of the transfection efficiency. Flow cytometric analysis of transfection efficiency in MM cell lines (A) as fluorescence intensity. B) Analysis, by qPCR, of miR-125b expression levels in MM cell lines transfected with miR125b. Values are mean ± standard deviation (SD), n = 3.

Figure S2. Cell viability modulation after miR-125b mimic and its modified analogs transfection. (A-C) MTS assay of MM cell lines transfected with 100 nM of miR-125b, miR-NC, miR-125b-Omet (Omet), miR-125b-LNA (LNA) or miR-125b-2'F (F). MM cell lines U266 (A), SKMM-1 (B) and RPMI 8226 (C) were analyzed after 24h and 48h from transfection. CTR = untreated cells used as control. Each experiment was repeated at least three times and the data are shown as a mean ± standard deviation (SD).

Figure S3. Evaluation of the transfection efficiency. Analysis, by qPCR, of miR-125b expression levels in U266 cells 48h after transfection with miR125b or its modified analogues. Values are mean ± standard deviation (SD), n = 3.

Figure S4. Densitometric analysis of Western Blot from Figure 3C/D. U266 cells transfected with 100 nM of miR-125b, miR-NC, miR-125b-Omet (Omet), miR-125b-LNA (LNA) or miR-125b-2'F (F) were collected for Western blotting analysis after 24h and 48 h from transfection. Densitometric analysis of IL6-R (A), cRaf (B), Akt (C), Bcl-2 (D), Mcl1 (E), STAT3 (F), pSTAT3 (G), EIF5A (H), 4EBP1 (I), p4EBP1 (L) and p53 (M) bands was performed with ImageJ analysis tool. Each column reflects the relative amount of the protein as a ratio of each protein band relative to the corresponding loading control. * $p \leq 0.05$; ** $p \leq 0.01$; *** $p \leq 0.001$ compared to miR-NC group.

Figure S5. Apoptosis evaluation by FACS analysis. U266 cell line was transfected with miR-125b, miR-NC or miR-125b-Omet at a final concentration of 100 nM for 24h (A) and 48h (B). After double staining with PI and Annessin V, as described in “Materials and Methods”, cells were analyzed by flow cytometric analysis. The data are representative of three different experiments that have always yielded similar results. Stacked bar histogram represents the percentage of cells in the four quadrants.

Figure S6. Flow cytometric analysis of autophagy. U266 cell line was transfected with miR-125b, miR-NC, miR-125b-Omet (Omet), miR-125b-LNA (LNA) or miR-125b-2'F (F) at a final concentration of 100 nM for 48h. After monodansylcadaverine (MDC) staining, cells were analysed on the BD Accuri C6™ flow cytometer. B and D) Replicates of the histograms reported in Fig. 4C of the main manuscript. A, C and E) Representative gating strategy of the three replicates.

Figure S7. Flow cytometric analysis of autophagy. U266 cell line was transfected with miR-125b, miR-NC, miR-125b-Omet (Omet), miR-125b-LNA (LNA) or miR-125b-2'F (F) at a final concentration of 100 nM for 72h. After monodansylcadaverine (MDC) staining cells were analysed on the BD Accuri C6™ flow cytometer. The histogram shows the relative intensity fluorescence intensity (MFI) with respect to control sample. The results are shown as means ± standard deviation (SD) for three independent experiments.

Figure S8. Analysis of senescence. Senescence-associated β-galactosidase assay. U266 cells were transfected with 100 nM of miR-125b, miR-NC, miR-125b-Omet (Omet), miR-125b-LNA (LNA) or miR-125b-2'F (F). After 72h from transfection the cells were incubated with staining solution as described in “Materials and Methods” and counted with a light microscope. The histogram shows the percent of blue cells. The results are shown as means ± standard deviation (SD) for three independent experiments.

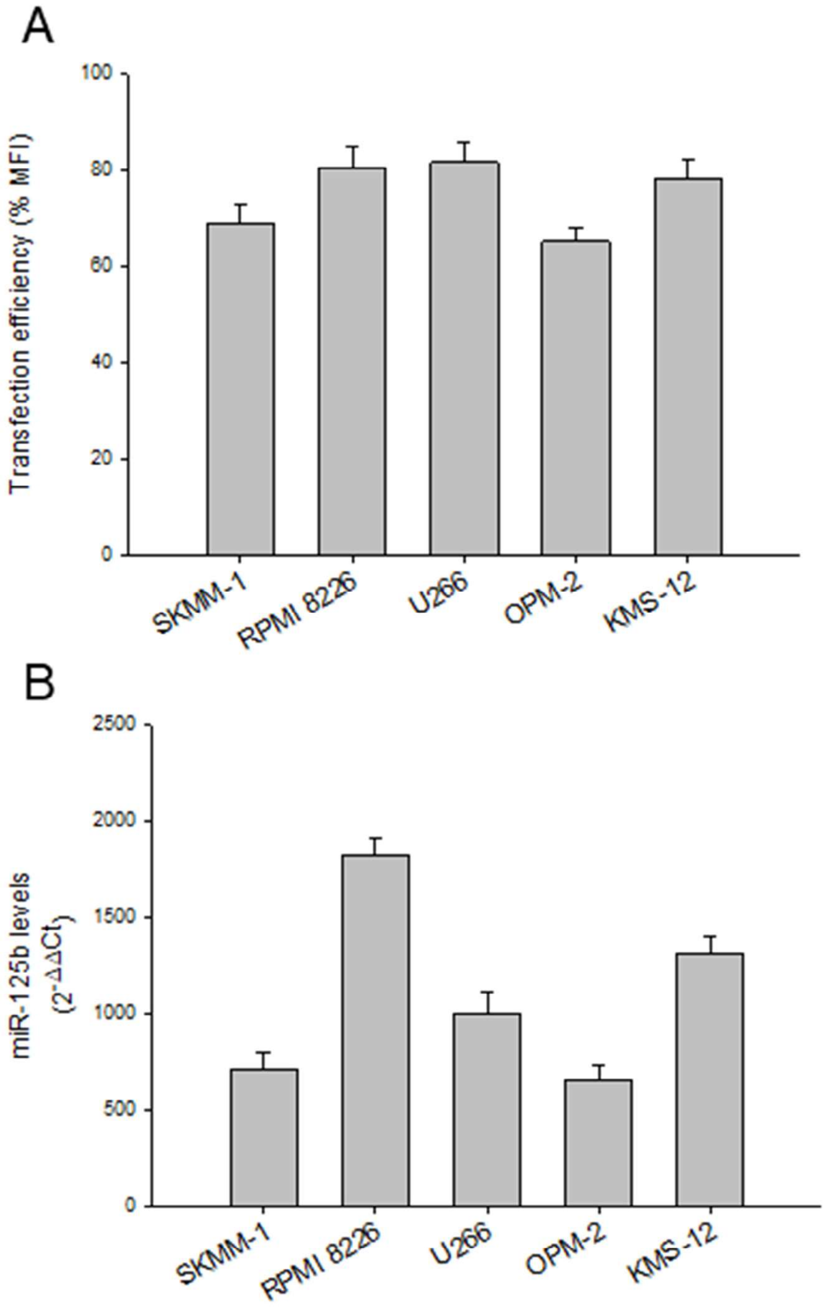
A
100
80
60
40
20
0
Transfection efficiency (% MFI)
SKMM-1
RPMI 8226
U266
OPM-2
KMS-12
B
2500
2000
1500
1000
500
0
miR-125b levels
($2^{-\Delta\Delta Ct}$)
SKMM-1
RPMI 8226
U266
OPM-2
KMS-12

Figure S1

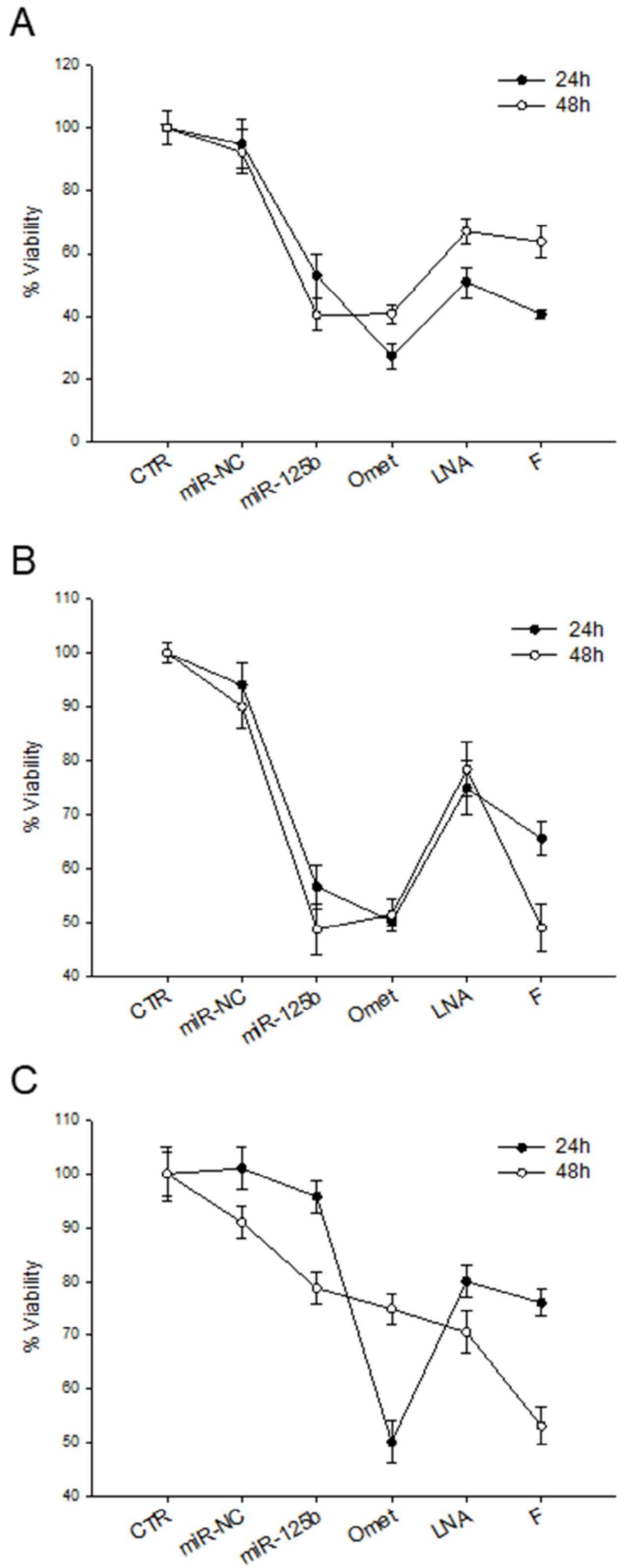
A
B
C
% Viability
24h
48h
CTR
miR-NC
miR-125b
Omet
LNA
F
0
20
40
60
80
100
120
50
70
90
110

Figure S2

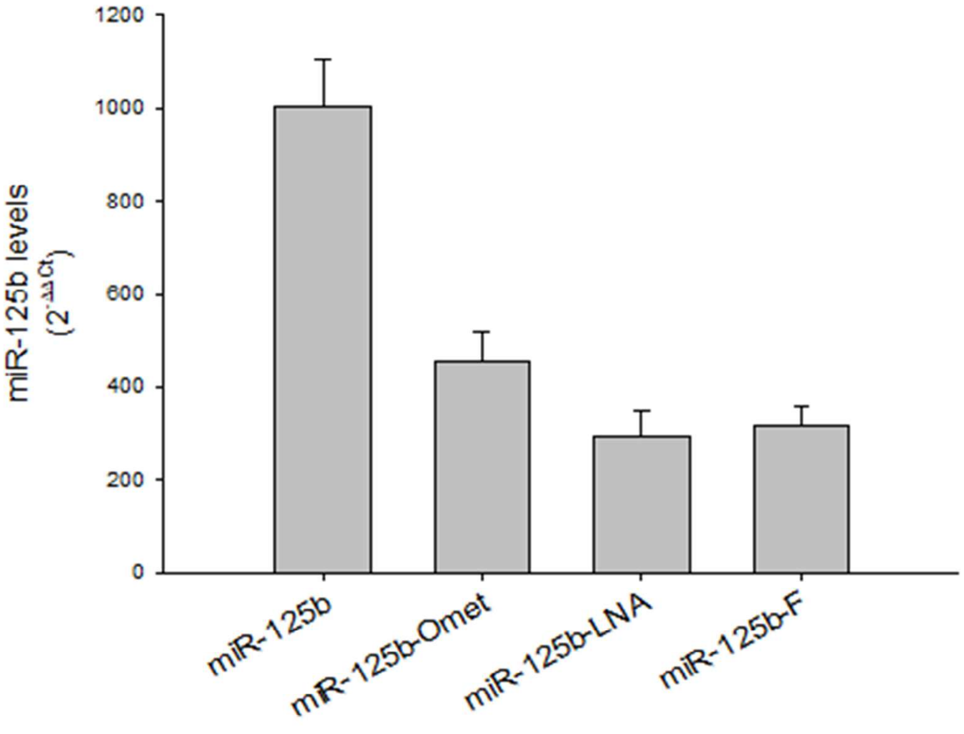
1200
1000
800
600
400
200
0
miR-125b levels
(2-ΔΔCt)
miR-125b
miR-125b-Omet
miR-125b-LNA
miR-125b-F

Figure S3

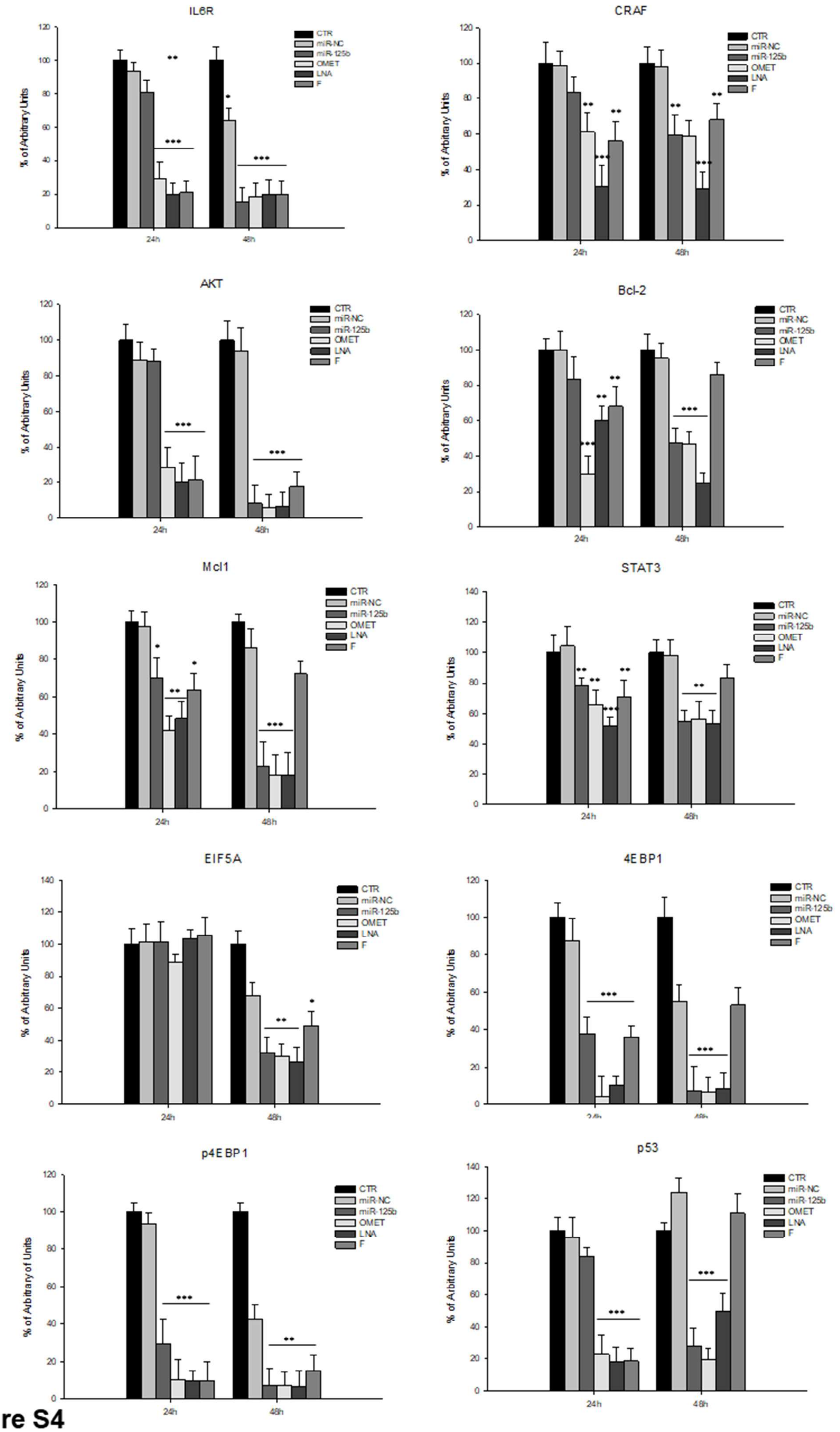
IL6R
CTR
miR-NC
miR-125b
OMET
LNA
F
% of Arbitrary Units
24h
48h
CRAF
AKT
Bcl-2
Mcl1
STAT3
EIF5A
4EBP1
p4EBP1
% of Arbitrary of Units
p53

Figure S4

A

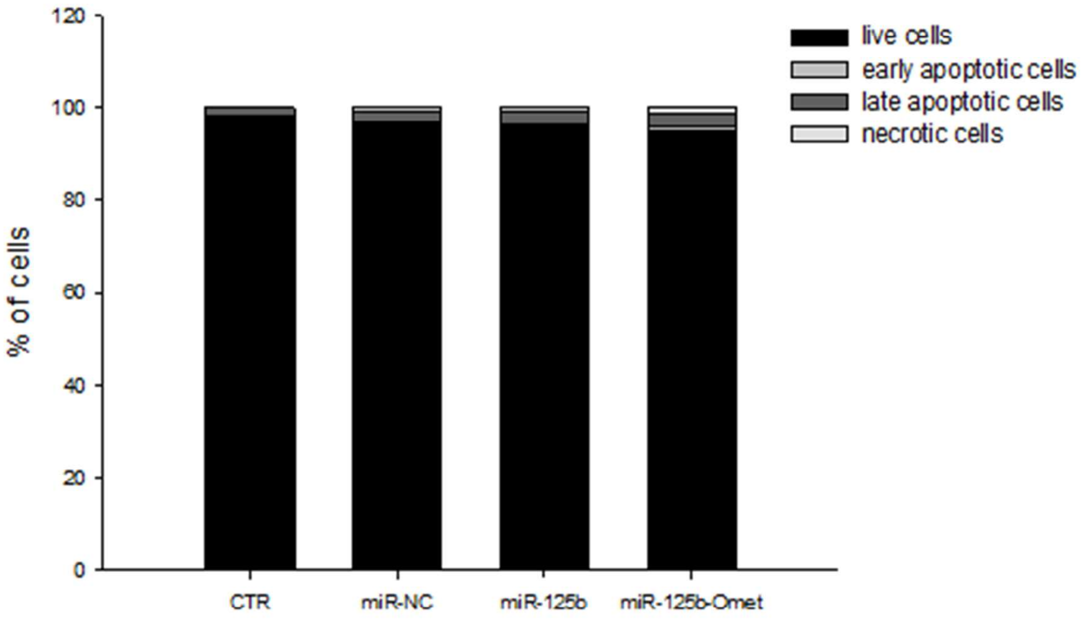

120
100
80
60
40
20
0
% of cells
live cells
early apoptotic cells
late apoptotic cells
necrotic cells
CTR
miR-NC
miR-125b
miR-125b-Omet

B

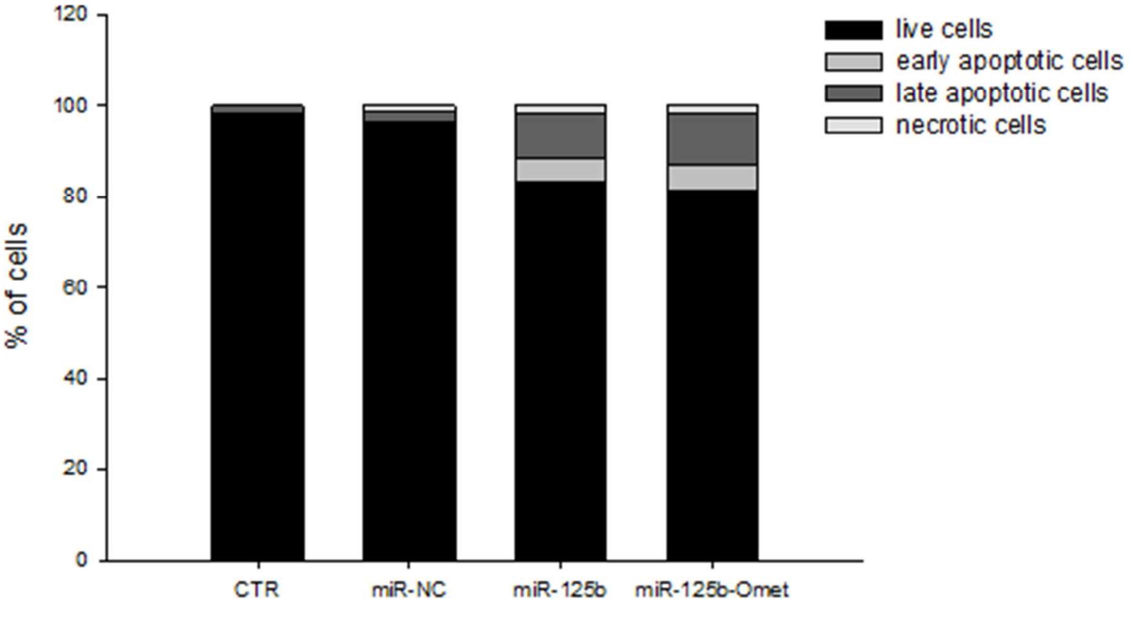

120
100
80
60
40
20
0
% of cells
live cells
early apoptotic cells
late apoptotic cells
necrotic cells
CTR
miR-NC
miR-125b
miR-125b-Omet

Figure S5

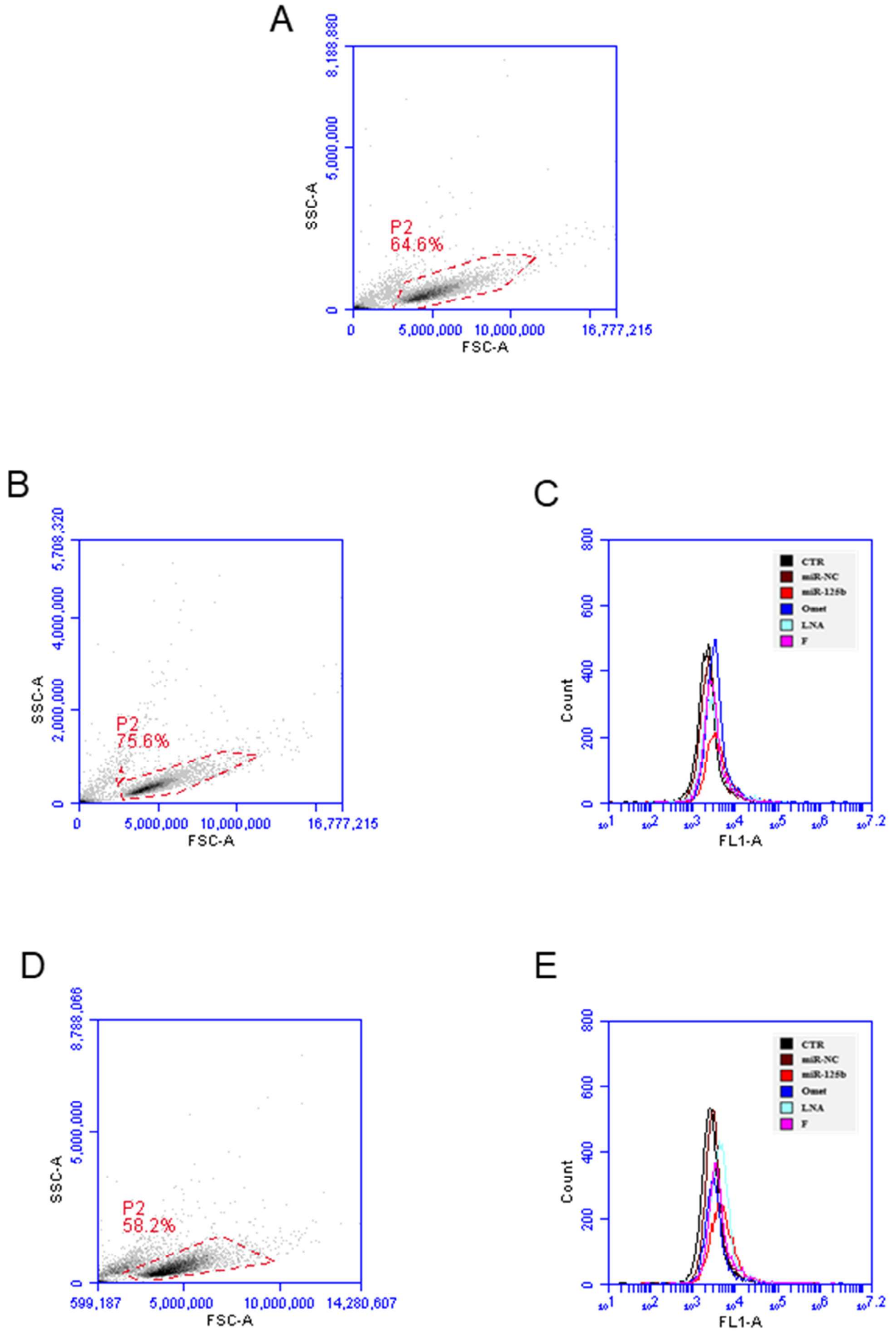
A
B
C
D
E
SSC-A
FSC-A
P2
64.6%
75.6%
58.2%
8,188,880
5,000,000
0
5,000,000
10,000,000
16,777,215
5,708,320
4,000,000
2,000,000
599,187
14,280,607
8,788,066
Count
FL1-A
800
600
400
200
CTR
miR-NC
miR-125b
Omet
LNA
F

Figure S6

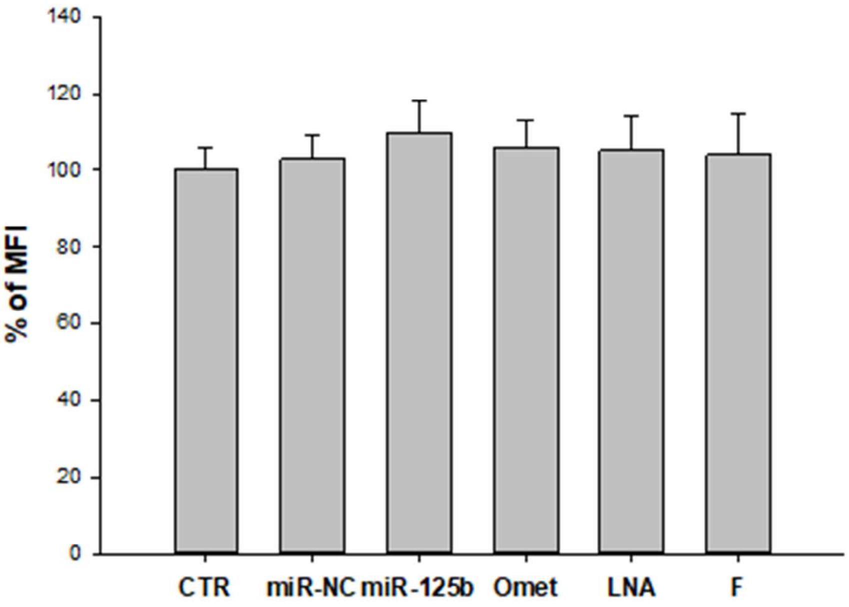

Figure S7

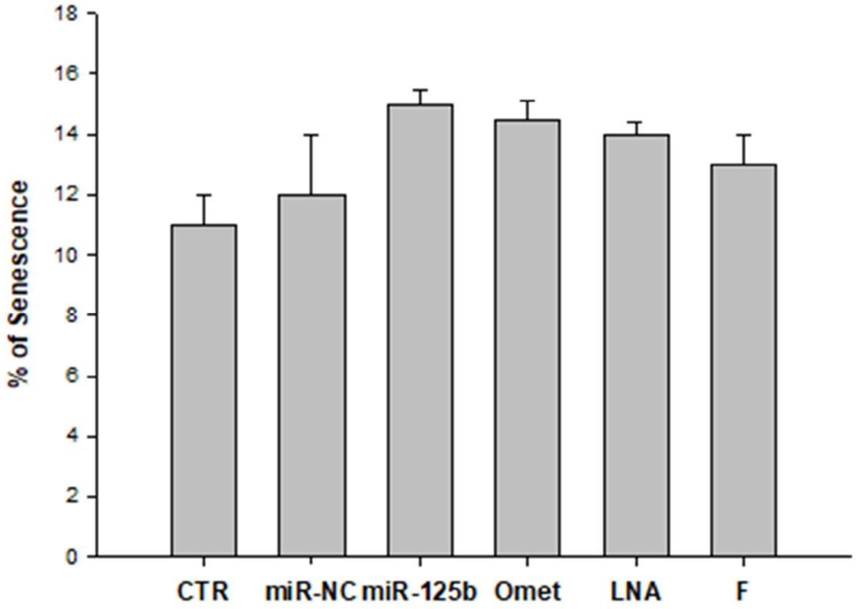

18
16
14
12
10
8
6
4
2
0
% of Senescence
CTR
miR-NC
miR-125b
Omet
LNA
F

Figure S8
